# An overview of the key routes to the best selling 5-membered ring heterocyclic pharmaceuticals

**DOI:** 10.3762/bjoc.7.57

**Published:** 2011-04-18

**Authors:** Marcus Baumann, Ian R Baxendale, Steven V Ley, Nikzad Nikbin

**Affiliations:** 1 Innovative Technology Centre, Department of Chemistry, University of Cambridge, Lensfield Road, CB2 1EW Cambridge, UK

**Keywords:** five-membered rings, heterocycles, medicinal chemistry, pharmaceuticals, synthesis

## Abstract

This review presents a comprehensive overview on selected synthetic routes towards commercial drug compounds as published in both journal and patent literature. Owing to the vast number of potential structures, we have concentrated only on those drugs containing five-membered heterocycles and focused principally on the assembly of the heterocyclic core. In order to target the most representative chemical entities the examples discussed have been selected from the top 200 best selling drugs of recent years.

## Introduction

Over the past few decades organic chemistry has seen tremendous progress and this has enabled the synthetic chemist to assemble virtually any molecular structure imaginable given reasonable time and sufficient resources. A steady increase in architectural complexity and the incorporation of more diverse molecular functionality has been a notable feature of pharmaceutical research and development. This general trend has emerged as a consequence of the better understanding of the genome and has resulted in many highly specific therapeutic targets being elucidated.

However, even today it might be argued that because of the perceived simpler structures of drug molecules when compared to complex natural products, only a limited repertoire of synthetic transformations are utilised for their construction. Furthermore, many of the modern pioneering developments in organic synthesis including new highly selective and mild bond forming reactions such as metathesis and C–H activation, asymmetric transformations as well as polymer- and technology-assisted syntheses are underused.

In order to evaluate the validity of this hypothesis we decided to investigate the syntheses of the best-selling pharmaceutical substances focusing not only on the type of transformations involved but more importantly on the way the heterocyclic components were assembled. Aromatic and non-aromatic heterocyclic rings are a predominant architectural constant of pharmaceuticals and allow for variable interactions with the biological target which are not possible using simpler carbocyclic motifs. Based on our observations, we will moreover be able to evaluate the degree these privileged structures utilise innovative and challenging synthetic strategies. In addition, it will be possible to establish which reactions are most frequently employed and which ones are surprisingly rare or notably absent. Furthermore, from this study it will be possible to judge whether novel methods and transformations developed within the academic community are commonly applied in the later stages of drug research.

In order to illustrate the diversity of synthetic methods used by the pharmaceutical industry to generate heterocycle containing molecules we decided in the first part of this review to focus mainly on five-membered aromatic heterocycles represented within the top 200 best selling drugs [1–2]. This review abstracts information available from many literature sources including patents to provide a selection of the most commonly used routes in drug synthesis. Different heterocyclic structures will be introduced and discussed in individual subsections. In Table 1 a summary of these structures is shown. We believe that this article will give an enlightening overview of both the classical heterocycle syntheses as well as interesting but less used transformations.

**Table 1:** Heterocylic structures discussed in this review.

Name	Label	Structure	Heterocycle core	

atorvastatin	**1**	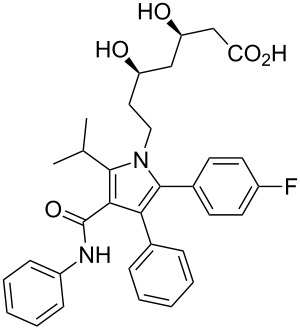	pyrrole	Figure 1
sunitinib	**36**	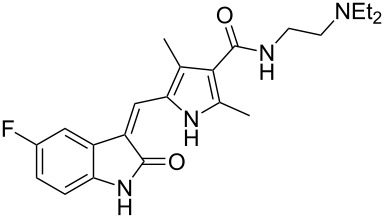	pyrrole	Scheme 7
sumatriptan	**49**	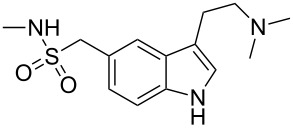	indole	Scheme 9
zolmitriptan	**50**	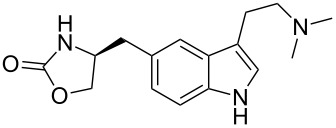	indole	Scheme 9
naratriptan	**69**	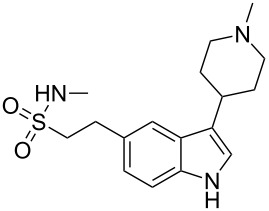	indole	Scheme 15
rizatriptan	**76**	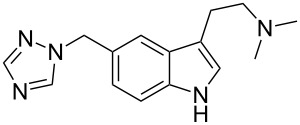	indole	Scheme 17
eletriptan	**87**	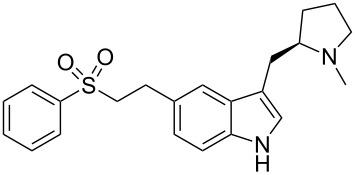	indole	Scheme 20
fluvastatin	**2**	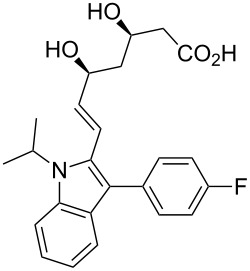	indole	Scheme 25
ondansetron	**119**	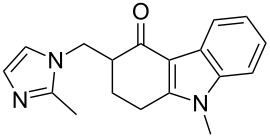	indole	Scheme 27
tadalafil	**132**	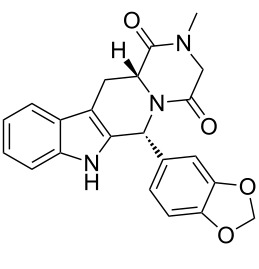	indole	Scheme 28
carvedilol	**136**	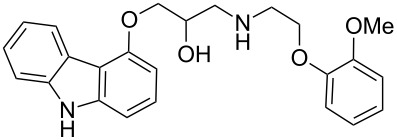	carbazole	Figure 4
etodolac	**153**	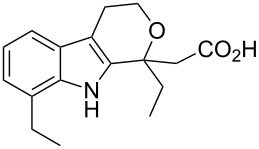	carbazole (analogue)	Scheme 31
losartan	**157**	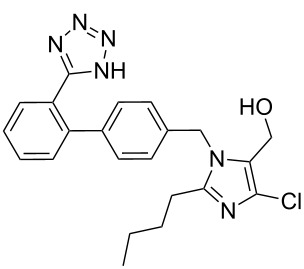	imidazole	Figure 5
olmesartan	**158**	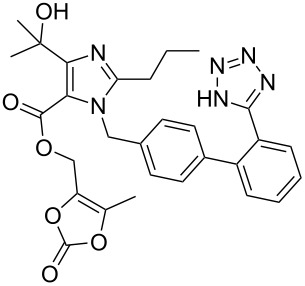	imidazole	Scheme 36
ondansetron	**119**	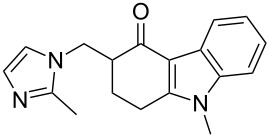	imidazole	Scheme 37
esomeprazole	**190**	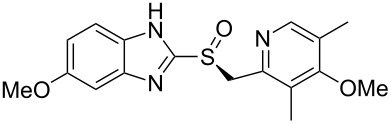	benzimidazole	Scheme 39
pantoprazole	**193**	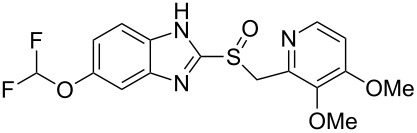	benzimidazole	Scheme 40
candesartan	**204**	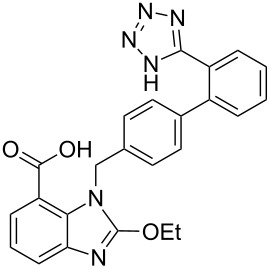	benzimidazole	Scheme 41
telmisartan	**217**	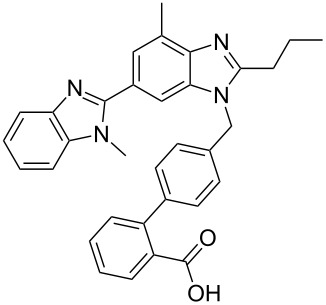	benzimidazole	Scheme 43
zolpidem	**227**	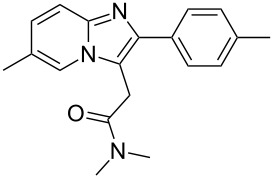	imidazopyridine	Scheme 45
celecoxib	**235**	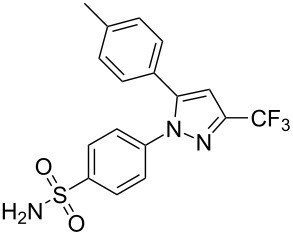	pyrazole	Figure 7
pazopanib	**246**	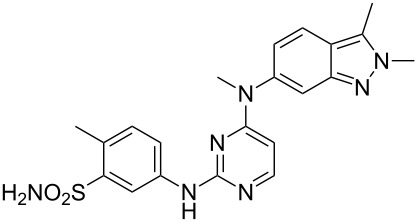	indazole	Scheme 50
anastrozole	**257**	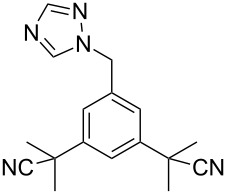	1,2,4-triazole	Scheme 51
rizatriptan	**76**	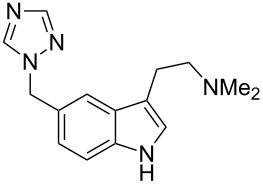	1,2,4-triazole	Scheme 51
letrozole	**256**	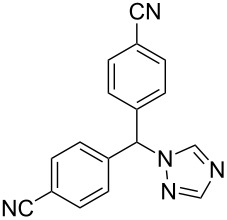	1,2,4-triazole	Scheme 51
sitagliptin	**275**	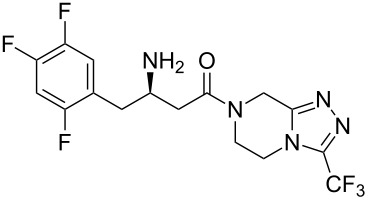	1,2,4-triazole	Scheme 55
maraviroc	**286**	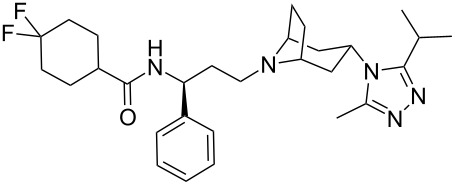	1,2,4-triazole	Scheme 57
alprazolam	**297**	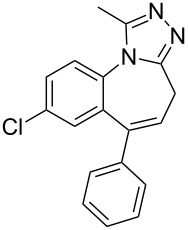	1,2,4-triazole	Scheme 58
itraconazole	**307**	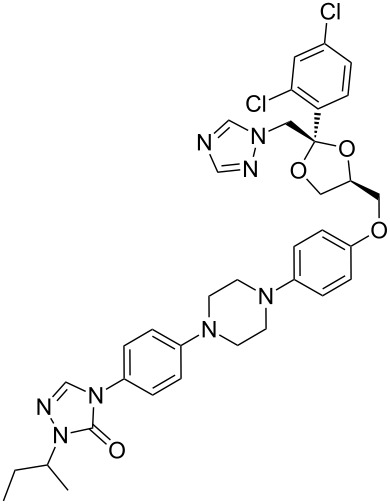	1,2,4-triazole	Figure 9
rufinamide	**315**	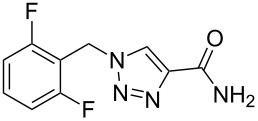	1,2,3-triazole	Scheme 61
valsartan	**319**	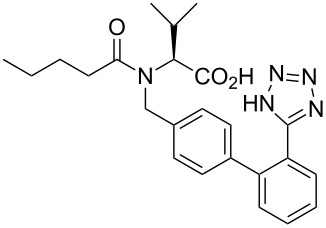	tetrazole	Scheme 62
losartan	**157**	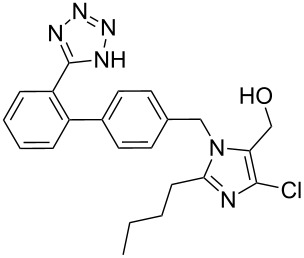	tetrazole	Scheme 63
cilostazole	**325**	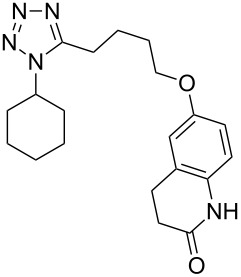	tetrazole	Scheme 64
cefdinir	**329**	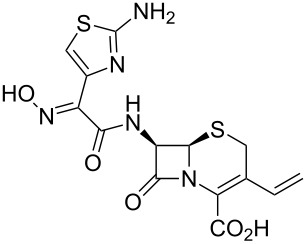	thiazole	Figure 11
ritonavir	**337**	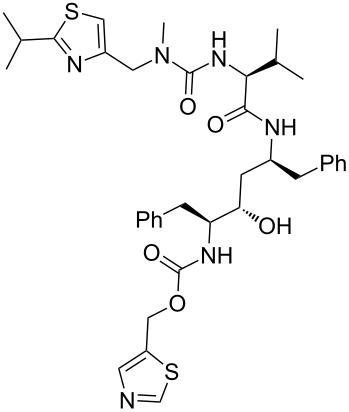	thiazole	Scheme 66
pramipexole	**345**	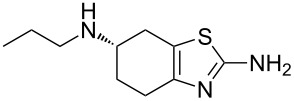	thiazole	Scheme 67
famotidine	**352**	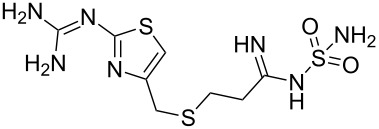	thiazole	Scheme 69
febuxostat	**359**	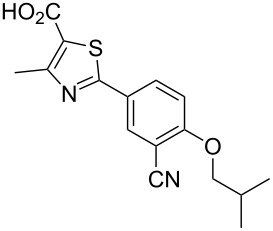	thiazole	Scheme 70
ziprasidone	**367**	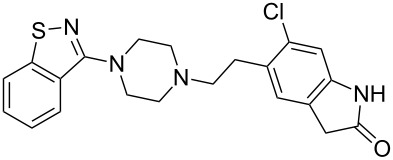	thiazole	Scheme 71
ranitidine	**377**	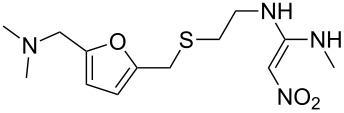	furan	Scheme 73
nitrofurantoin	**382**	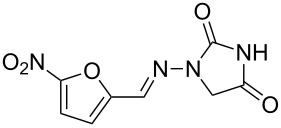	furan	Scheme 74
amiodaron	**385**	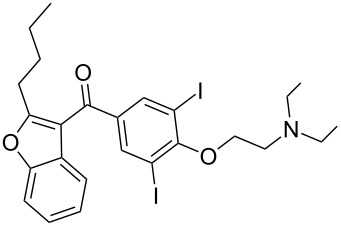	furan	Scheme 76
raloxifene	**392**	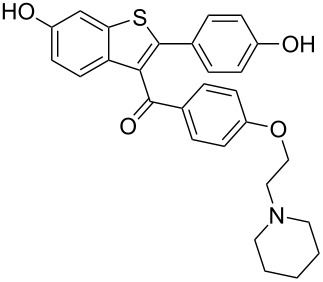	thiophene	Scheme 77
olanzapine	**399**	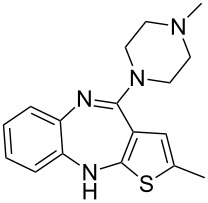	thiophene	Scheme 79
clopidogrel	**420**	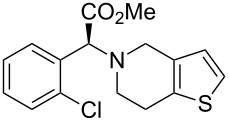	thiophene	Scheme 81
timolol	**432**	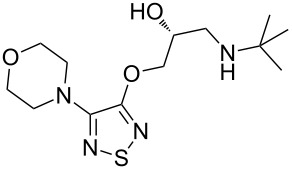	thiadiazole	Figure 14
tizanidine	**440**	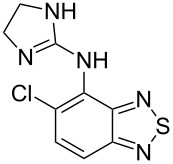	thiadiazole	Scheme 85
leflunomide	**446**	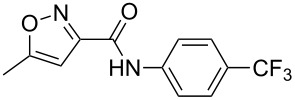	isoxazole	Scheme 86
sulfamethoxazole	**447**	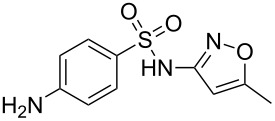	isoxazole	Scheme 87
risperidone	**454**	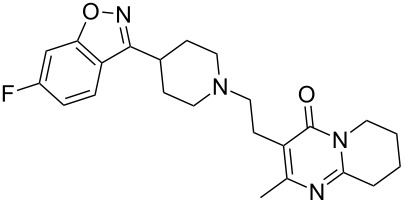	benzoisoxazole	Scheme 88

## Review

### Pyrroles

**Figure 1:**
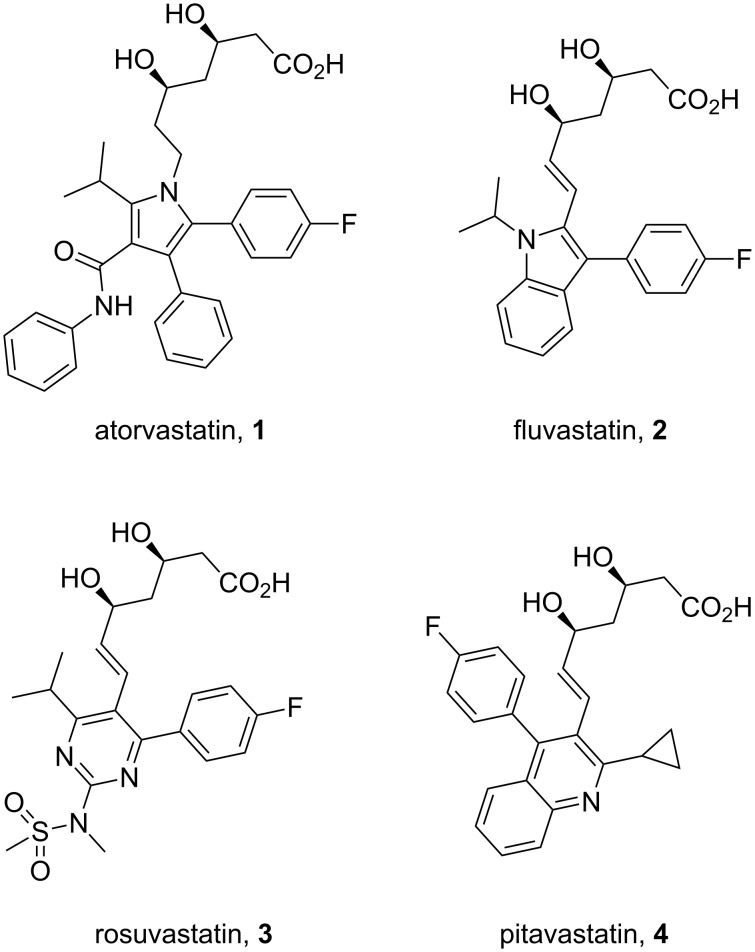
Structures of atorvastatin and other commercial statins.

Pyrrole, a five-membered nitrogen containing heterocycle is present in some of the most common biologically important molecules, i.e., chlorophyll and haem. However, while the pyrrole ring is not widely represented in pharmaceutical compounds, the core structure of atorvastatin (**1**, Lipitor; Figure 1), the best-selling drug substance of the last few years, does contain a penta-substituted pyrrole ring. This drug is an example of a competitive HMG-CoA-reductase inhibitor belonging to the 7-substituted 3,5-dihydroxyheptanoic acid family. In atorvastatin this important syn-1,3-diol moiety is connected to the other functional constituents through a fully substituted pyrrole ring instead of the more elaborate systems which are encountered in other members of this family.

**Figure 2:**
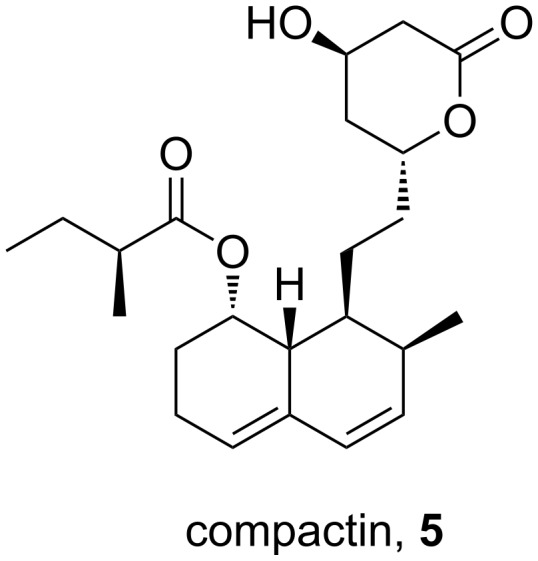
Structure of compactin.

The initial synthesis of atorvastatin was reported by the Warner–Lambert company [3]. From structure–activity relationship (SAR) studies it was found that the addition of substituents at the 3- and 4-position of the pyrrole scaffold significantly increases potency when compared to the more classical 2,5-disubstituted pyrroles. This study culminated in the discovery of atorvastatin which has a five times greater potency then the initial lead, the fungal metabolite compactin (**5**, Figure 2).

**Scheme 1:**
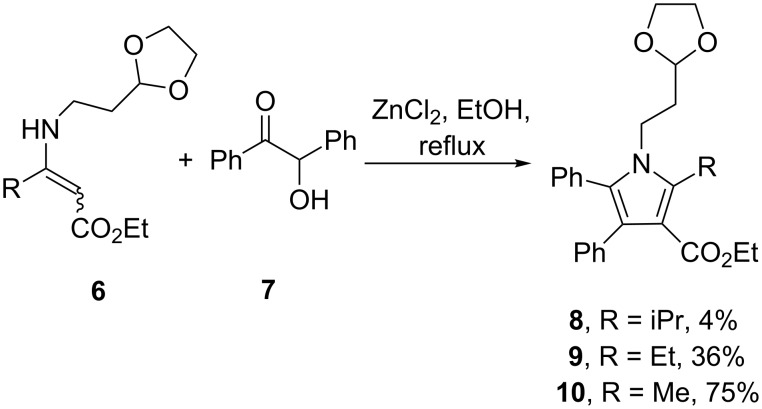
Synthesis of pentasubstituted pyrroles.

Although it was possible to construct such fully substituted pyrrole rings by ZnCl_2_-catalysed condensation reactions between a functionalised enamine **6** and simple benzoin (**7**), this method proved to be less successful for more complex pyrroles of which atorvastatin was an example (Scheme 1).

**Scheme 2:**
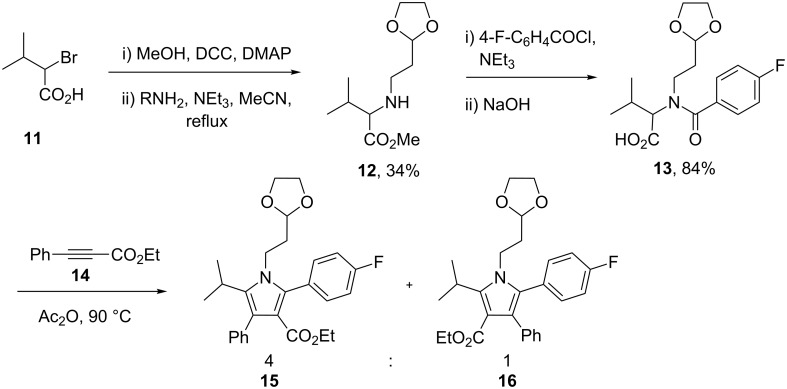
[3 + 2] Cycloaddition to prepare 5-isopropylpyrroles.

In order to prepare the 5-isopropylpyrrole derivative **16** a more efficient [3 + 2] cycloaddition of an acetylene component with an amido acid **13** was developed. Unfortunately, reacting ethyl phenylpropiolate with the corresponding amido acid in hot acetic anhydride afforded a 4:1 mixture of regioisomers with the major product being the undesired regioisomer (Scheme 2).

**Scheme 3:**
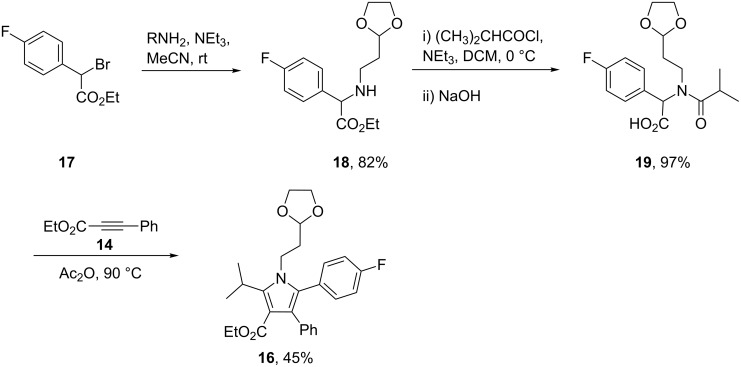
Regiospecific [3 + 2] cycloaddition to prepare the pyrrole scaffold.

However, the analogous reaction with isomer **19** was found to be completely regiospecific leading to the formation of the desired product **16** albeit in only moderate yield (Scheme 3).

**Scheme 4:**
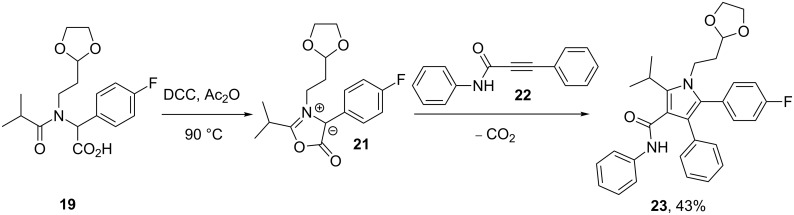
Formation of the pyrrole core of atorvastatin via [3 + 2] cycloaddition.

By modification of the coupling partners a more defined dipolar [3 + 2] cycloaddition between *N*,3-diphenylpropynamide (**22)** and an in situ generated mesoionic species **21** furnished the desired product **23** regiospecifically [4] (Scheme 4).

**Scheme 5:**
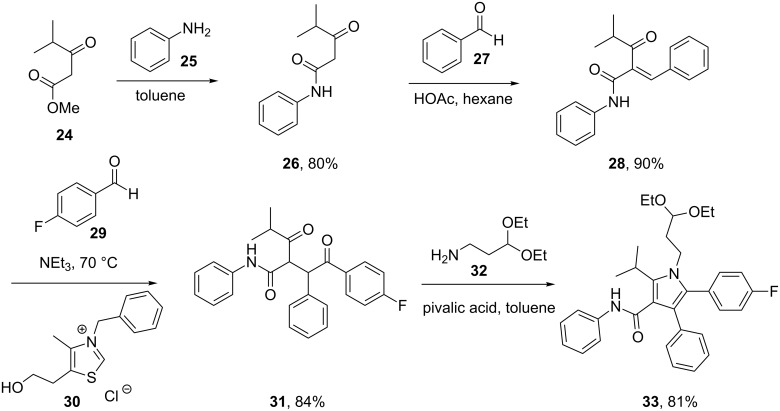
Formation of pyrrole **33** via the Paal–Knorr reaction.

A further synthetic approach was based on the classical Paal–Knorr cyclocondensation of a highly substituted 1,4-diketone with a primary amine bearing a masked aldehyde functionality. The 1,4-diketone component, which can be accessed via a 3-step sequence [5] starting from aniline, was refluxed with 3-aminopropionaldehyde diethylacetal **32** in toluene under mildly acidic conditions to afford the fully substituted pyrrole motif in 81% isolated yield following crystallisation [6]. The key transformation in this sequence is a thiazolium-mediated Stetter reaction between 4-fluorobenzaldehyde (**29**) and an advanced Michael acceptor obtained from an initial Knoevenagel condensation (Scheme 5).

**Scheme 6:**
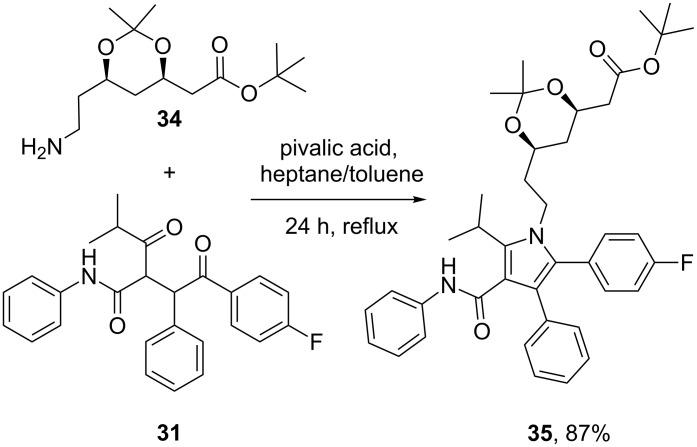
Convergent synthesis towards atorvastatin.

In order to improve the overall yield as well as the convergency, the industrial route [7] introduced the fully elaborated side chain **34** by condensation with the previously described 1,4-diketone **31** (Scheme 5 and Scheme 6). The desired atorvastatin structure was then obtained in only three additional steps via acetal cleavage, ester hydrolysis and formation of the calcium salt.

**Figure 3:**
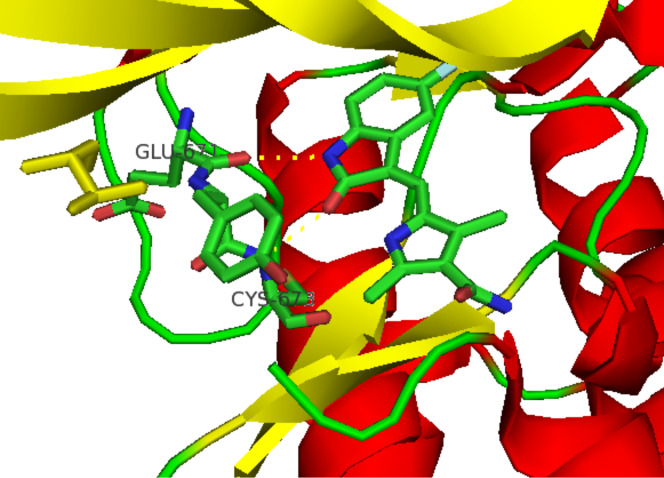
Binding pocket of sunitinib in the TRK KIT.

Sunitinib (**36**, Sutent) is Pfizer’s novel receptor tyrosine kinase inhibitor (RTK) approved in 2006 by the FDA for the treatment of both renal cell carcinoma and gastrointestinal stromal tumors. This drug has been suggested as a second-line therapy for patients developing mutation-related resistance to other cancer medications such as imatinib (Gleevec). Sunitinib binds to the inactivated, auto-inhibited conformation of the KIT kinase by partially occupying the space normally filled by the ADP’s adenine in the phosphorylated protein. The indolinone section is located in a deeper pocket with the heteroatoms being involved in H-bonding glutamate and tyrosine residues whilst the pyrrole ring and the diethylaminoethyl appendage are exposed to the solvent environment [8] (Figure 3).

**Scheme 7:**
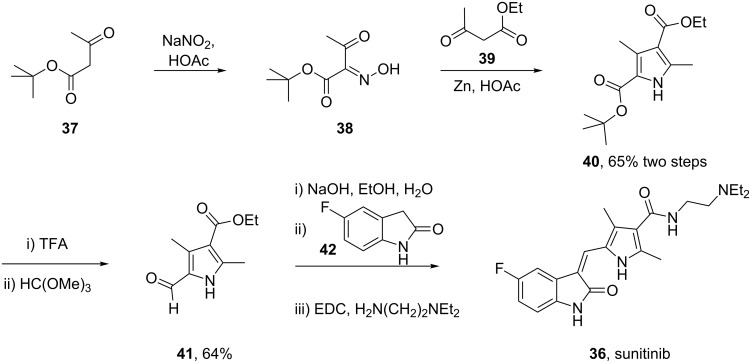
Synthesis of sunitinib.

Structurally, this medication consists of a complex pyrrole-substituted 2-indolinone core which can be prepared by a late stage aldol condensation between a 2-indolinone fragment **42** with the corresponding pyrrole aldehyde **41** (Scheme 7). The oxime functionality in compound **38**, which is obtained by nitrosation of *tert-*butyl acetoacetate (**37**) [9], is reduced by zinc to give an unstable aminoketone intermediate. Subsequent enamine formation and ring closure affords the fully substituted pyrrole ring **40**. Selective deprotection of the *tert*-butyl ester with concomitant decarboxylation yields ethyl 2,4-dimethylpyrrole-3-carboxylate which can be formylated at the free ring position by trimethyl orthoformate to yield the fully elaborated pyrrole aldehyde (**41**). The aforementioned aldol condensation unites both key fragments: Ester hydrolysis and amide formation complete the synthesis (Scheme 7).

**Scheme 8:**
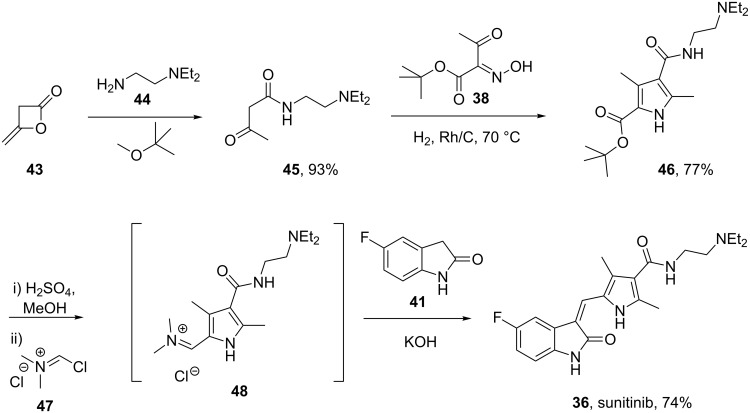
Alternative synthesis of sunitinib.

An attractive alternative synthesis of sunitinib introduces the amide side chain earlier by ring opening of 4-methyleneoxetan-2-one (**43**) with *N*,*N-*diethylethane-1,2-diamine (**44**) [10]. The resulting β-keto amide **45** is then converted to the analogous pyrrole by condensation with the previously mentioned oxime **38** under reductive conditions. Deprotection and decarboxylation of the remaining *tert*-butyl ester produces the desired pyrrole intermediate, which upon treatment with Vilsmeier reagent undergoes a formal acylation. This product is not isolated but reacted directly with **41** in an aldol condensation to yield sunitinib (Scheme 8).

### Indoles

**Scheme 9:**
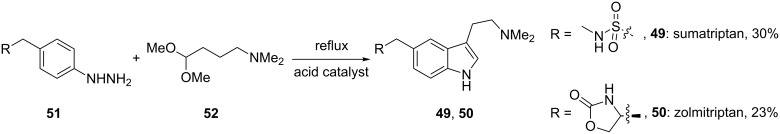
Key steps in the syntheses of sumatriptan and zolmitriptan.

The neuroamine transmitter serotonin contains an indole ring, so it is not surprising that indoles are a recurring theme in many drugs affecting central nervous system (CNS) function including antidepressants, antipsychotics, anxiolytics and antimigraine drugs, as well as psychedelic agents. Indole is also one of the best represented heterocyclic motifs present in the top selling pharmaceuticals, being found in eight of the top 200 drugs, with five of these belonging to the triptan family of antimigraine treatments. The classical Fischer indole synthesis is usually reported as one of the first choice routes to prepare these scaffolds. Drugs such as GSK’s serotonin receptor modulators sumatriptan (**49**, Imitrex) and zolmitriptan (**50**, Zomig) use the Fischer indole synthesis at a late stage in order to form the desired compound albeit in only low to moderate yields (Scheme 9).

**Scheme 10:**
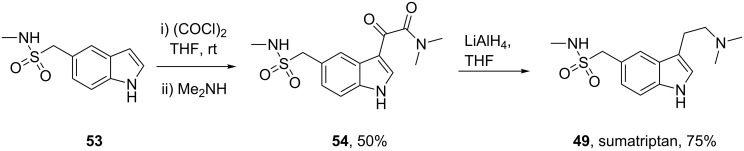
Introduction of the *N*,*N*-dimethylaminoethyl side chain.

However, in sumatriptan the indole product resulting from the Fischer synthesis can still react further which leads to the formation of by-products and significantly reduced yields. One way to minimise this was to protect the nitrogen of the sulfonamide group prior to indole formation [11]. This leads not only to an increased yield in the indole forming step (to 50%) but also facilitates chromatographic purification. The dimethylamino group can be present from the beginning of the synthesis or can be introduced via displacement of chloride or reduction of a cyano moiety. Alternatively, the dimethyl ethylene amine side chain can be introduced in position 3 via a Friedel–Crafts-type acylation. The resulting acid chloride is transformed in situ to the corresponding amide which on reduction with lithium aluminium hydride affords sumatriptan (Scheme 10) [12].

**Scheme 11:**
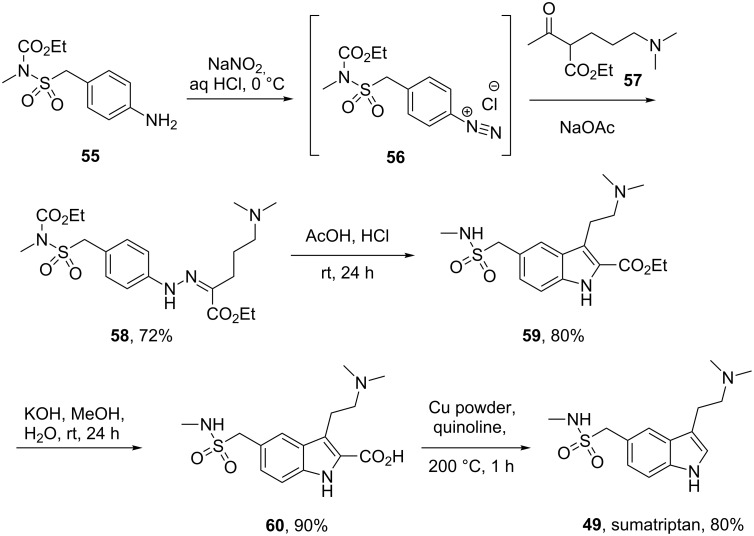
Japp–Klingemann reaction in the synthesis of sumatriptan.

In the standard Fischer indole synthesis a hydrazine, which is most commonly derived from the corresponding diazonium salt, is reacted with a suitable carbonyl compound. Alternatively, the Japp–Klingemann reaction can be used to directly couple the diazonium salt with a β-ketoester to obtain a hydrazone which can then undergo indole ring formation (Scheme 11) [13].

As can be seen from Scheme 11 the indole **59** prepared via the Japp–Klingemann reaction is substituted at position 2 by an ester group which prevents reaction with electrophiles, thereby reducing the amount of undesired by-products. A simple sequence of hydrolysis and decarboxylation then affords sumatriptan [14].

**Scheme 12:**
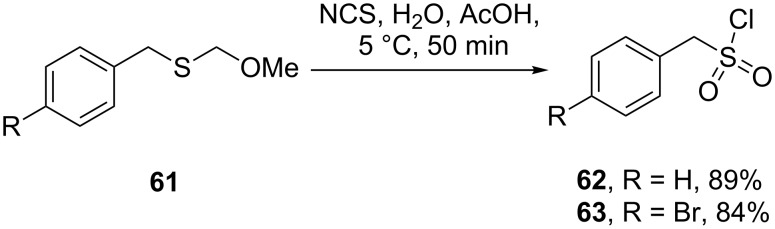
Synthesis of the intermediate sulfonyl chlorides **62** and **63**.

All the reported methods for the synthesis of sumatriptan begin with the sulfonamide group already present on the aromatic ring and several routes are possible to introduce this functional group. The scalable route to the sulfonamides inevitably involves the preparation of the sulfonyl chloride intermediate which is then trapped with the desired amine. The sulfonyl chloride can also be prepared from the corresponding hemithioacetal **61** by treatment with NCS in wet acetic acid (Scheme 12). This efficient oxidation produces only methanol and formaldehyde as by-products [15].

**Scheme 13:**
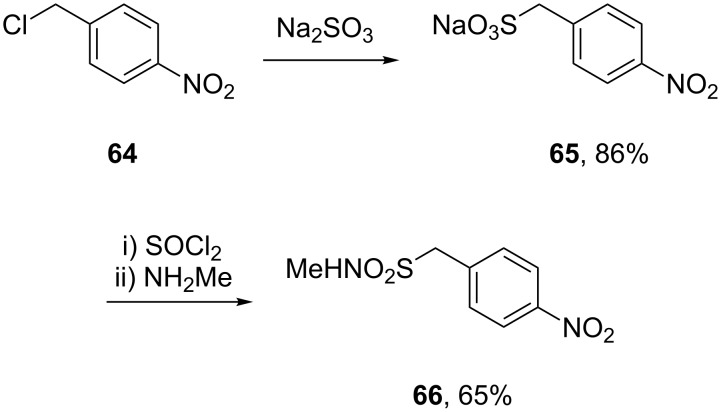
Alternative introduction of the sulfonamide.

Another possible approach is based on the direct displacement of a benzylic chloride by sodium sulfite and subsequent sulfonamide formation as shown in Scheme 13 [16].

**Scheme 14:**
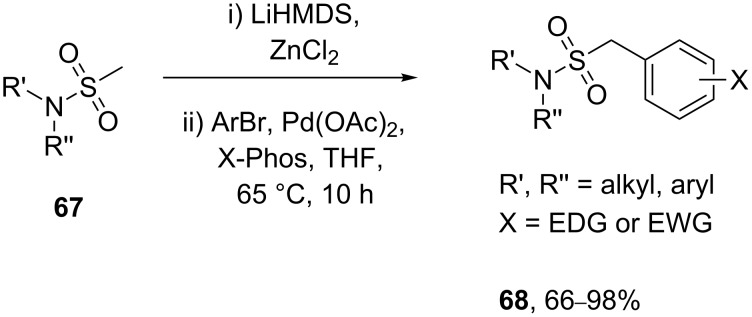
Negishi-type coupling to benzylic sulfonamides.

A more recent method utilises a palladium-catalysed Negishi coupling to access a diverse library of benzylic sulfonamides, all prepared in high yields. As this route employs sulfonamide-stabilised anions the preparation and handling of unstable sulfonyl chloride intermediates is therefore circumvented (Scheme 14) [17].

**Scheme 15:**
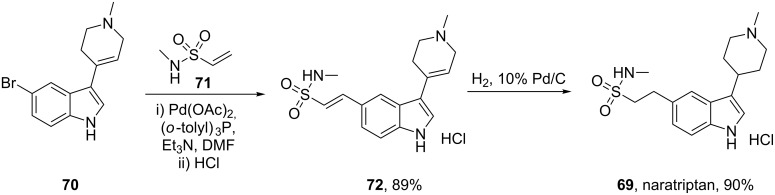
Heck reaction used to introduce the sulfonamide side chain of naratriptan.

An analogous Heck reaction approach has also been employed to introduce a homologous side chain as demonstrated during the assembly of the antimigraine drug naratriptan (**69**, Amerge, Scheme 15) [18].

**Scheme 16:**
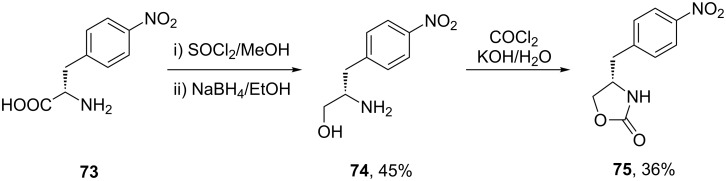
Synthesis of the oxazolinone appendage of zolmitriptan.

Another related antimigraine drug developed by GSK is zolmitriptan (Zomig, **50**). The indole ring and its substitution at the 3-position are identical to that of sumatriptan, however, the sulfonamide side chain has been replaced by an oxazolinone ring. The original synthesis utilised 4-nitro-*L*-phenylalanine (**73**) as the precursor for the oxazoline moiety (Scheme 16) [19]. The resulting Fischer indole synthesis is similar to that used for sumatriptan (Scheme 9).

More recent reports [20] deal with improvements to both these syntheses making them more economic and environmentally benign. For example, the three steps to zolmitriptan, namely, diazonium salt formation, its subsequent reduction and Fischer indole synthesis can all be carried out in aqueous media without the need for intermediate isolation. It has also been demonstrated that the reduction of the initially formed diazonium salt can be accomplished by using sodium metabisulfite (Na_2_S_2_O_5_) rather than using the more toxic stannous chloride or the less water soluble and more expensive sodium sulfite.

**Scheme 17:**
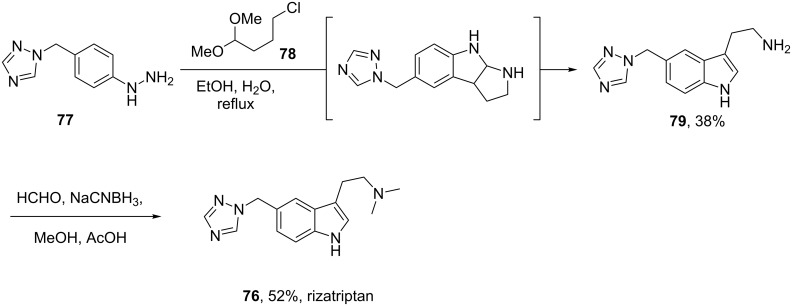
Grandberg indole synthesis used in the preparation of rizatriptan.

Preparation of the indole ring system in the antimigraine drug rizatriptan (**76**, Maxalt) makes use of the Grandberg variation of the Fischer indole synthesis as the final stage reaction to form the tryptamine-type moiety, via formation and re-opening of a tricyclic intermediate [21–22]. Although not a high yield process, this one pot sequence establishes the indole ring and the pendant primary amine group in a single operation (Scheme 17).

**Scheme 18:**
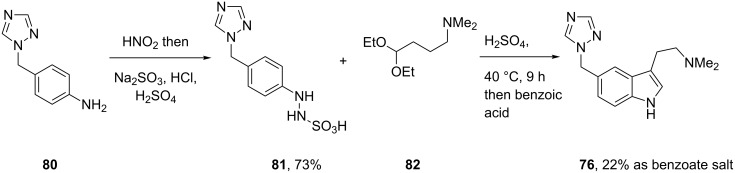
Improved synthesis of rizatriptan.

However, the main disadvantage of this process is the need for high temperatures which leads to the formation of dimeric impurities and results in the requirement for extensive chromatographic purification. An improved process which circumvents the formation of such dimeric impurities involves the reduction of the intermediate diazonium salt with sodium sulfite and hydrochloric acid to form an easily isolable and crystalline phenylhydrazine sulfonic acid **81**. The subsequent Fischer indole synthesis can then be performed cleanly at much lower temperatures (Scheme 18) and yields pure rizatriptan as its benzoate salt [23]. In addition, the use of precursor **82**, already possesing the desired dimethylamino group, simplifies the reaction sequence and tunes the reactivity of the amine preventing it from participating in many side reactions.

**Scheme 19:**
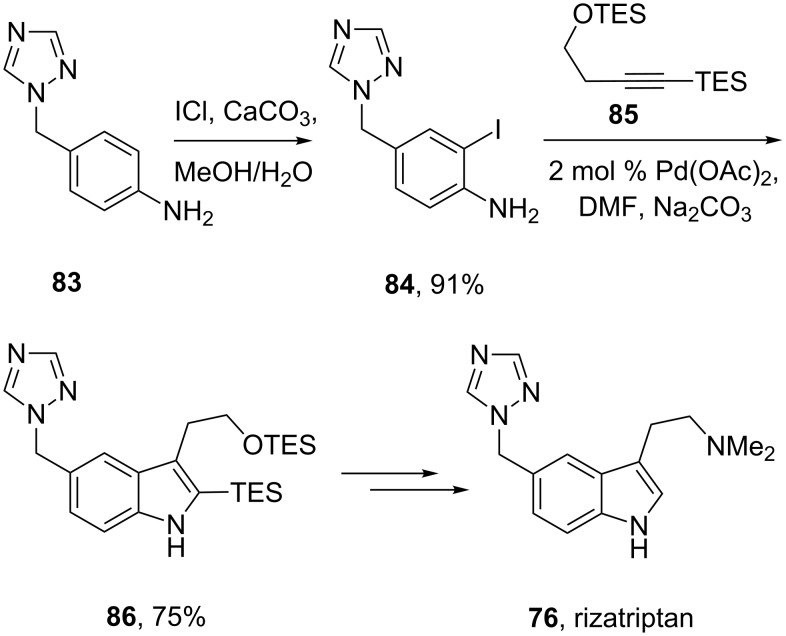
Larock-type synthesis of rizatriptan.

The indole core of rizatriptan can also be prepared by a palladium-catalysed coupling first reported by Larock [24]. The iodoaniline derivative **84** required for this approach can be readily synthesised from triazolomethyl aniline **83** by treatment with iodine monochloride in aqueous methanol. The *bis*-TES-protected butynol **85** was found to be the most efficient coupling partner and led smoothly to the desired indole **86**. Subsequent removal of the TES-groups and introduction of a dimethylamino moiety furnishes the desired drug compound [25–27] (Scheme 19).

**Scheme 20:**
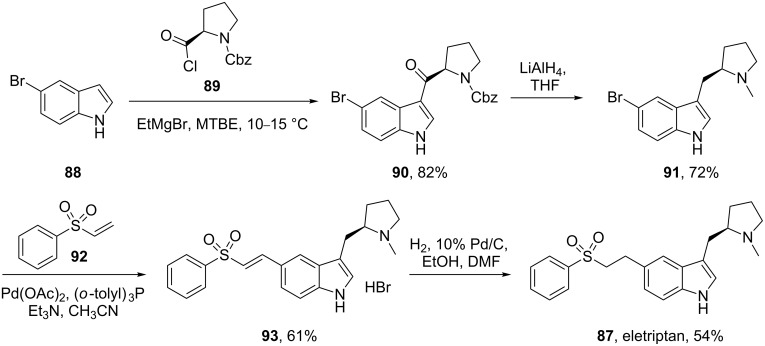
Synthesis of eletriptan.

Eletriptan (**87**, Relpax) is yet another indole-containing antimigraine drug. A process route for the synthesis of eletriptan published by Pfizer starts from a preformed bromo-indole **88** [28] (Scheme 20). In order to perform the acylation of the indole ring on larger scale, ethylmagnesium bromide and the corresponding acid chloride **89** are added concurrently from two different sides of the reactor to stop these reagents reacting with each other. This method of adding the reagents circumvents the necessity to isolate the magnesium salt of the indole and increases the yield from 50 to 82%. The carbonyl group of the proline side chain is then reduced simultaneously with the complete reduction of the Cbz-group to a methyl group with lithium aluminium hydride. Finally, the sulfonate side chain is introduced via a Heck-type coupling similar to that of naratriptan (Scheme 15), followed by hydrogenation of the double bond to afford eletriptan (Scheme 20).

**Scheme 21:**
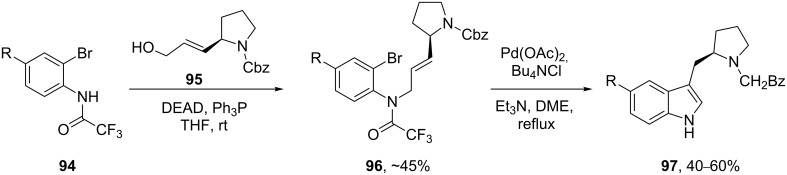
Heck coupling for the indole system in eletriptan.

A rather ingenious Mitsunobu coupling reaction has been used to create a highly functionalised substrate **96** for an intramolecular Heck reaction resulting in a very short and succinct synthesis of eletriptan and related analogues **97** [29] (Scheme 21).

**Scheme 22:**
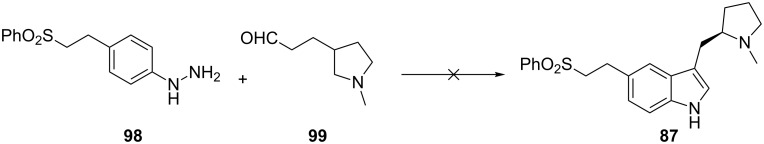
Attempted Fischer indole synthesis of elatriptan.

Interestingly, it was found that the most obvious approach, the direct Fischer indole synthesis, to prepare the core of eletriptan as shown in Scheme 22 is not successful [30]. This is believed to be due to the instability of the phenyl hydrazine species **98** under the relatively harsh reaction conditions required to promote the cyclisation.

**Scheme 23:**
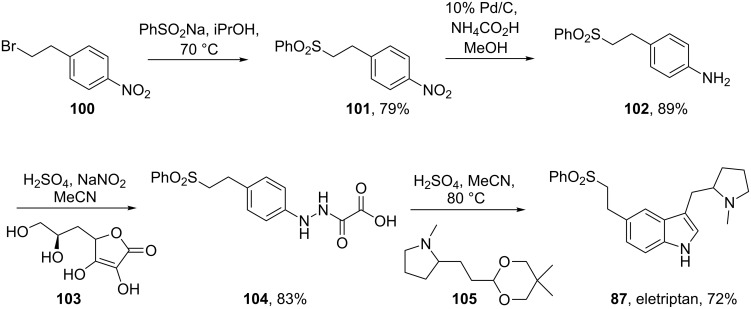
Successful Fischer indole synthesis for eletriptan.

However, this problem could be avoided by using an acid-labile oxalate protected hydrazine **104** as depicted in Scheme 23. The yield of this step can be further improved up to 84% if the corresponding calcium oxalate is used.

**Scheme 24:**
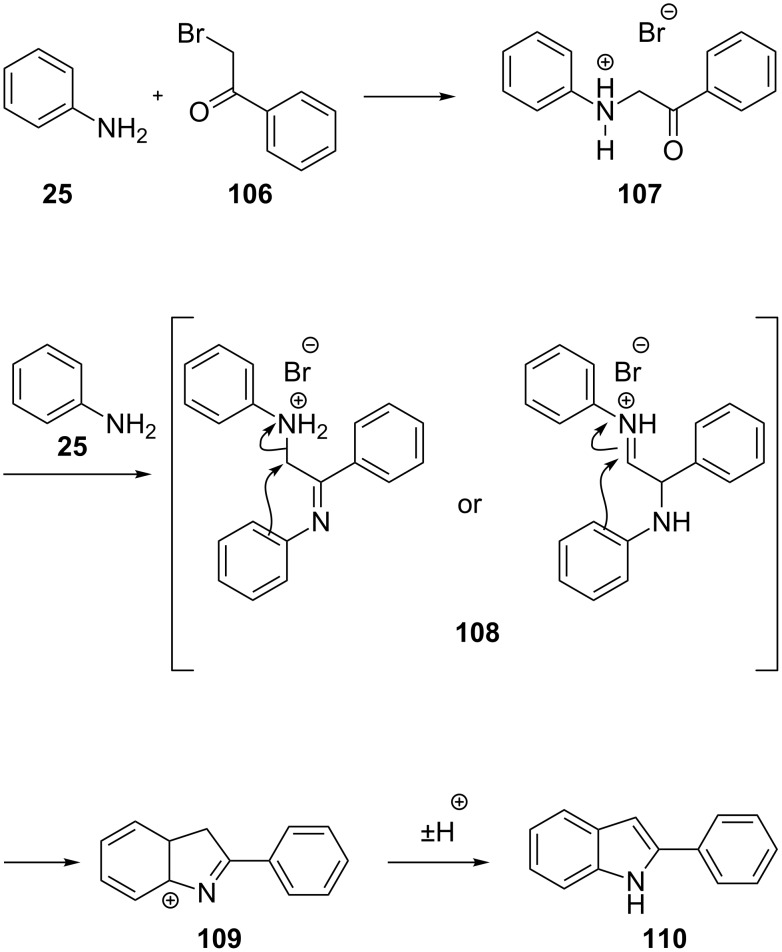
Mechanistic rationale for the Bischler–Möhlau reaction.

The Bischler–Möhlau reaction is an alternative indole synthesis employing an α-bromo ketone and an excess of aniline to give a 2-arylindole derivative **110** [31] (Scheme 24). For a long time this procedure has received little attention due to the requirement for rather extreme reaction conditions. However, the use of microwave radiation in combination with Lewis acid catalysis allows the reaction to be conducted much more efficiently [32]. This gives mid range yields (50–70%) of the 2-arylindoles over the two step sequence and tolerates a selection of functional groups on the aniline ring.

**Scheme 25:**
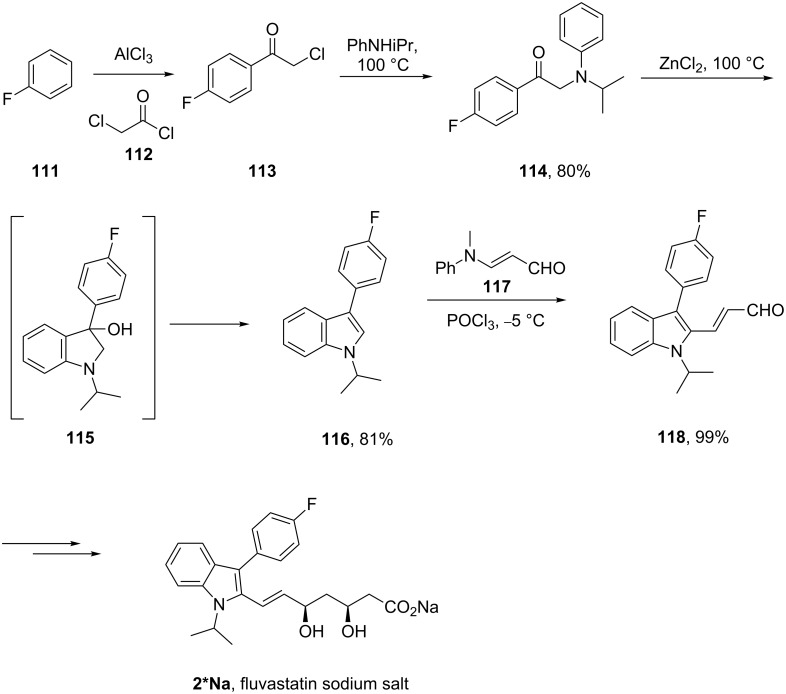
Bischler-type indole synthesis used in the fluvastatin sodium synthesis.

Fluvastatin (**2**, Lescol), a HMG-CoA reductase inhibitor was initially prepared by a Fischer indole synthesis [33] (Scheme 25). However, in the development stages it was decided that a Bischler–Möhlau type reaction could be used instead. In this case a ZnCl_2_-mediated Bischler-type indole synthesis [34] is used with stoichiometric amounts of the aniline which leads to the required 3-substituted indole core **116** at an early stage in the synthesis. A novel way to introduce a formyl substituent at the 2-position of the indole was subsequently developed to aid the introduction of the *syn*-diol pendant side-chain.

**Scheme 26:**
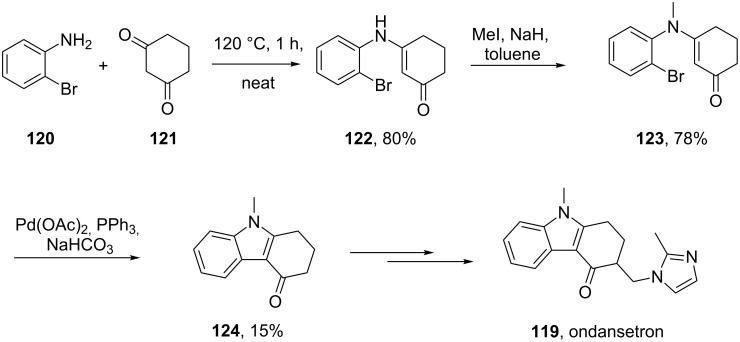
Palladium-mediated synthesis of ondansetron.

A completely different strategy was used in the synthesis of the serotonin 5-HT_3_ receptor antagonist ondansetron (**119**, Zofran). In this synthesis a palladium-catalysed intramolecular Heck-reaction was used to build the tricyclic indole core in a short and concise sequence (Scheme 26) [35–36].

**Scheme 27:**
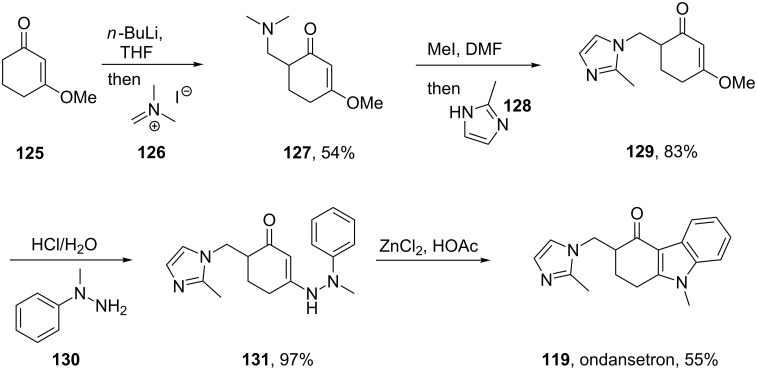
Fischer indole synthesis of ondansetron.

Alternatively, a direct Fischer indole synthesis between phenylmethyl hydrazine and a cyclic 1,3-dione derivative could be utilised to prepare the desired fully substituted tricyclic core of ondansetron (Scheme 27) [37].

**Scheme 28:**
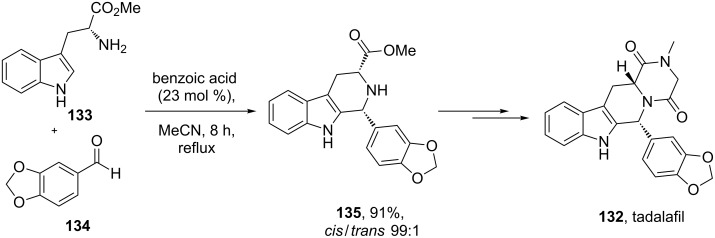
Optimised Pictet–Spengler reaction towards tadalafil.

A different approach was used in the synthesis of the phosphodiesterase inhibitor tadalafil (**132**, Cialis) starting from commercially available (D)-tryptophan methyl ester to form the indolopiperidine motif **135** via a Pictet–Spengler reaction followed by a double condensation to install the additional diketopiperazine ring (Scheme 28) [38–39].

To achieve the high levels of *cis* selectivity required from the Pictet–Spengler reaction, an extensive investigation of solvents and the influence of additives was undertaken [40]. It was identified that the use of a specific 23 mol % of benzoic acid significantly increased the *cis*/*trans* ratio from a base level of 55:45 to 92:8 (16 h reaction time at ambient temperature) in an overall yield of 86%. It was also determined that more polar solvents such as acetonitrile and nitromethane preferentially solvated the *trans* product and thereby allowed the isolation of the *cis* compound by precipitation. It was also shown that by heating the reaction mixture under reflux the product distribution could be driven to the thermodynamically more favoured *cis* isomer having both the ester and the piperonyl moiety in equatorial positions. Hence, after heating under reflux for 8 h the *cis*/*trans* ratio was found to be 99:1 and the product could be isolated in an overall yield of 91%. This work represents an impressive example of a well considered and executed process optimisation study.

### Carbazoles

Carvedilol (**136**, Coreg) is a general non-selective β-blocker, used in the treatment of mild to moderate congestive heart failure. The structure comprises of a core carbazole ring that plays an important role in its increased activity.

**Figure 4:**
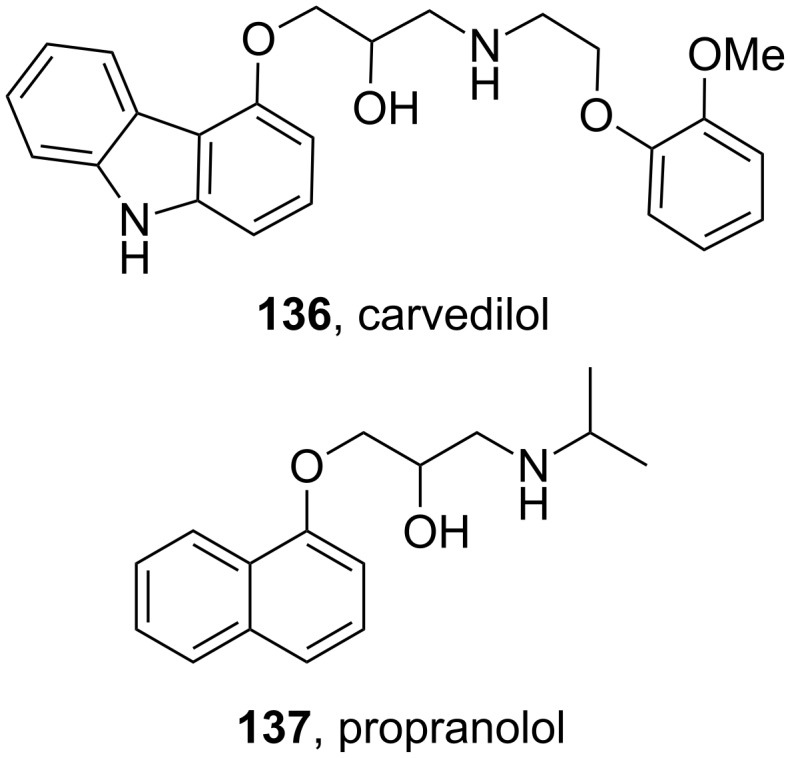
Structures of carvedilol **136** and propranolol **137**.

The cardioprotective effect of β-adrenergic blockers is attributed to their ability to reduce the myocardial workload by reducing the system’s requirement for oxygen. However, the activity of carvedilol is greater when compared to other members of the β-blocker family such as propranolol (**137**, Figure 4) implying that carvedilol has an additional antioxidant mode of action. It has been proposed that the carbazole ring may be involved in scavenging oxygen radicals thereby accounting for reduced myocardial damage [41].

**Scheme 29:**
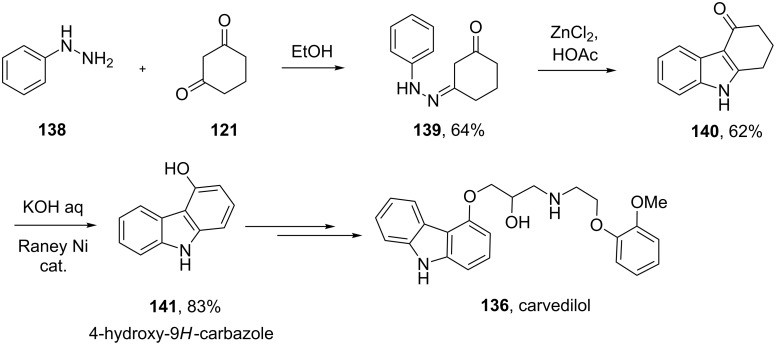
Synthesis of the carbazole core of carvedilol.

Since carbazoles are similar to indoles, analogous methods can be used for their synthesis. The key intermediate for carvedilol is 4-hydroxy-9*H*-carbazole (**141**) [42]. In analogy to the Fischer indole synthesis, cyclohexane-1,3-dione monophenyl hydrazone **139** is prepared via condensation of phenylhydrazine (**138**) with 1,3-cyclohexanedione (**121**, Scheme 29). This compound can then undergo an acid catalysed Fischer indole synthesis to yield tetrahydro-4-oxocarbazole **140**. Various methods have been described to dehydrogenate this intermediate including the use of bromine, sulfur, LiCl/CuCl, lead dioxide, chloranil or palladium on charcoal. However, the need to use these reagents in equal stoichiometry or even excess has led to the search for a new approach. It was found that a catalytic quantity of Raney nickel in an aqueous potassium hydroxide solution gives the desired 4-hydroxy compound **141** cleanly and in high conversion (Scheme 29).

**Scheme 30:**
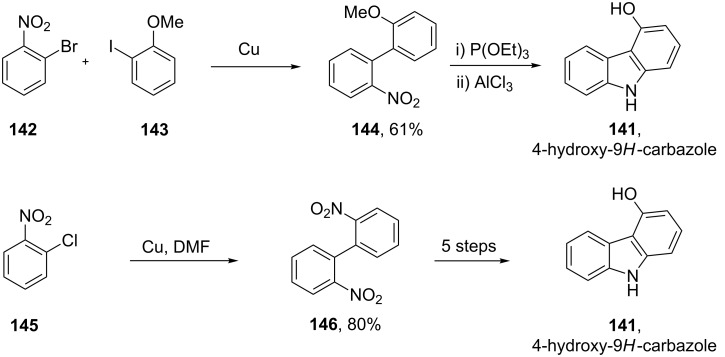
Alternative syntheses of 4-hydroxy-9*H*-carbazole.

Alternative routes to this key intermediate such as Ullmann-type reactions have also been reported, however, these usually rely on longer reaction sequences and require more expensive starting materials (Scheme 30) [42–43].

**Scheme 31:**
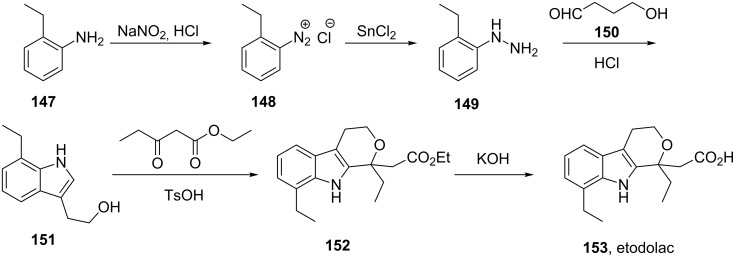
Convergent synthesis of etodolac.

Etodolac (**153**, Lodine) is a racemic non-steroidal anti-inflammatory tetrahydrocarbazole derivative used to treat inflammation and prescribed for general pain relief. Two general routes have been described for the preparation of this drug. In the first route the hydrazine **149** is prepared via the reduction of diazonium salt **148** with tin(II) chloride and subjected to a Fischer indole reaction with aldehyde **150**. The resulting indole is then condensed with ethyl 3-oxopropanoate followed by saponification of the ester to yield etodolac (Scheme 31) [44].

**Scheme 32:**
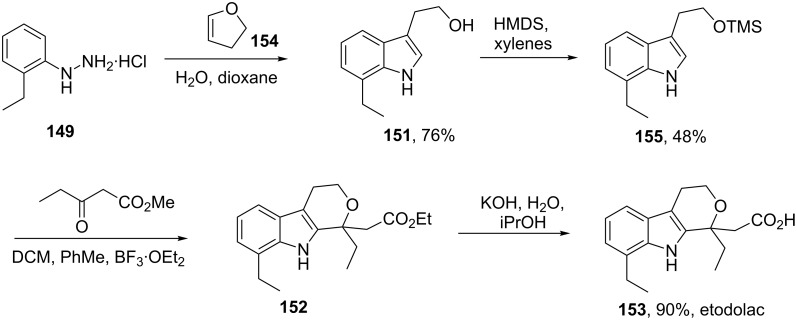
Alternative synthesis of etodolac.

The second described route [45–46] is similar but starts with a more readily available carbonyl surrogate; 2,3-dihydrofuran **154.** The Fischer indole reaction provides a primary alcohol which is TMS-protected and condensed with methyl 3-oxopentanoate under Lewis acid conditions. The use of the temporary silyl mask was found beneficial as it circumvents the need for chromatographic purification. Finally, simple saponification furnishes etodolac (Scheme 32).

Extensive biological studies have found that the majority of the reactivity of etodolac is derived from the (*S*)-(+)-enantiomer. The resolution of the racemate can be readily accomplished using cinchonine or the glucose derivative *N*-methyl-D-glucamine. The latter provides the pure (*S*)-(+)-enantiomer in 25.3% yield (>96% de) after two crystallisations. In addition, the racemisation of the undesired enantiomer via methyl ester formation and treatment with sodium hydroxide was found to be feasible allowing efficient recycling of this material [47].

### Imidazoles

Imidazole is an important biological building block being present in the amino acid histidine and possessing inherent catalyst and acid–base functionality. An imidazole ring is also a component of the biogenic amine histamine. It is interesting to note that the imidazole ring does not however appear in the most common H1 and H2 antagonists, presumably due to its metabolic vulnerability, with cimetidine being an exception. The use of such compounds has now been almost completely superseded by the prescription of alternative proton pump inhibitors such as esomeprazole.

**Figure 5:**
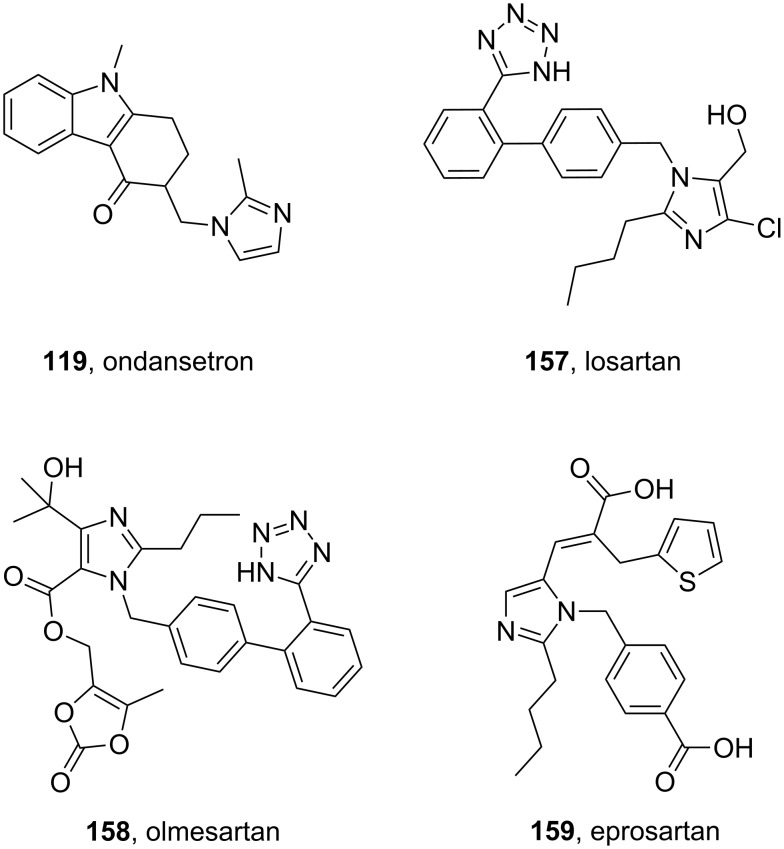
Structures of imidazole-containing drugs.

Imidazole containing drugs can be subdivided into two classes: monocyclic imidazoles and benzimidazoles. The former is represented by three drugs targeting hypertension losartan (**157**, Cozaar), olmesartan (**158**, Benicar), eprosartan (**159**, Eprozar) as well as nausea (ondansetron (**119**, Zofran)) (Figure 5). All of these drugs aptly highlight a different synthetic approach to the imidazole core. Early antagonists of the angiotensin II receptor were of a peptidic nature, suffered from poor bioavailability and also showed some agonistic activities. The first non-peptide antagonists were developed in the early 80’s. Although these compounds were selective for the AT_2_ receptor, they bound only weakly to their target protein. On examining the angiotensin II amino acid sequence, researchers took notice of an acidic residue on the NH_2_ terminus. Consequently, a carboxylic acid moiety was added to the designed ligand improving affinity for the AT_2_ receptor. However, the polar nature of the carboxylic acid group caused this compound to suffer from poor absorption and low bioavailability. At this stage a classical bioisostere exchange, i.e., replacing a carboxylic acid group with a tetrazole ring, was performed which resulted in increased lipophilicity [48] and the development of the orally active losartan.

In the absence of a crystal structure, mutation studies have shown that the AT_2_ antagonists such as losartan bind to an active site located within the membrane-bound part of the receptor, which is different to that of the peptide agonist. Further studies led to the discovery of valsartan, where the imidazole ring of losartan is replaced by an *N*-acylated amide which is suggested to mimic the C-terminus of angiotensin II [49].

**Scheme 33:**
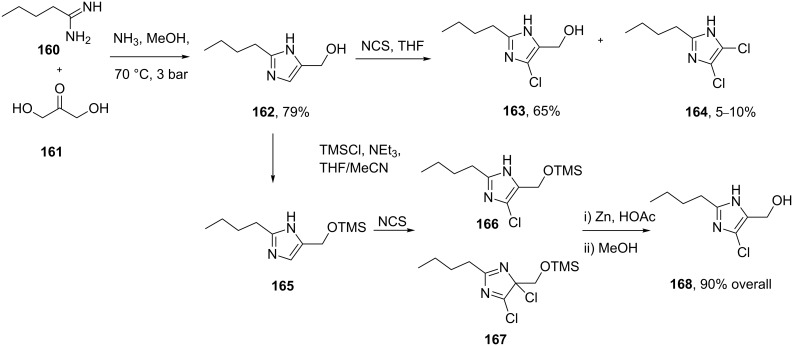
Synthesis of functionalised imidazoles towards losartan.

The imidazole ring of losartan, an antihypertensive and angiotensin II blocker is formed in a condensation reaction between valeroamidine **160** and dihydroxyacetone [50]. It was found that direct chlorination of the imidazole **162** also forms the dichlorination product **164** (as shown in Scheme 33) with formaldehyde as a by-product which proved difficult to suppress and made purification of the reaction mixture problematic. Hence, a sequence involving silyl protection, chlorination and deprotection was established which gave the desired product in 90% overall yield (Scheme 33).

**Scheme 34:**
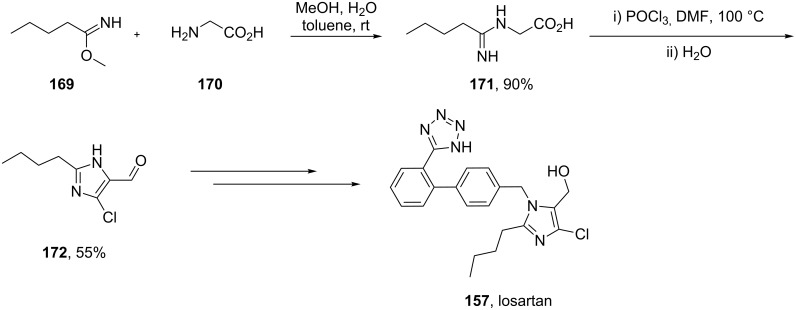
Direct synthesis of the chlorinated imidazole in losartan.

Alternatively, glycine can be reacted with methyl pentanimidate **169** to form the corresponding amidine **171** in high yield. Cyclisation, followed by a Vilsmeier-type reaction then furnishes the key chloroimidazolyl building block **172** in good yield (Scheme 34) [51].

**Scheme 35:**
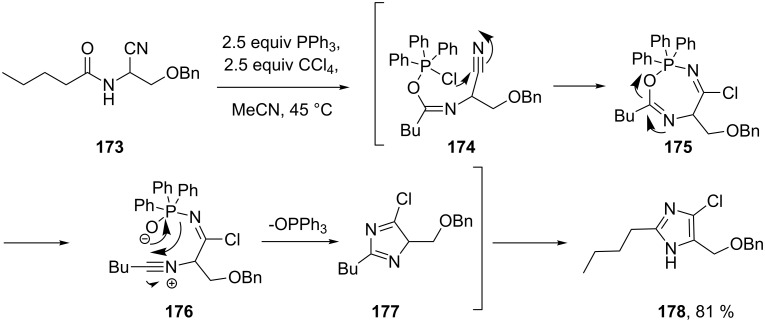
Synthesis of trisubstituted imidazoles.

More recently, Zhong and co-workers [52] reported a highly efficient one-pot procedure starting from *N*-acylated α-aminonitriles **173**. The desired 2,4,5-trisubstituted imidazole core **178** is formed in high yield in the presence of carbon tetrachloride and triphenylphosphine (Scheme 35). Mechanistic studies showed that the related imidoyl compounds do not themselves undergo ring closure to form imidazoles and it was therefore proposed that the reaction between carbon tetrachloride and triphenylphosphine to generate dichlorotriphenylphosphorane and (dichloromethylene)triphenylphosphorane was pivotal. The amidonitrile substrate **173** can then react with both species to form seven-membered cyclic intermediate **175** which collapses to form the imidazole compound.

**Scheme 36:**
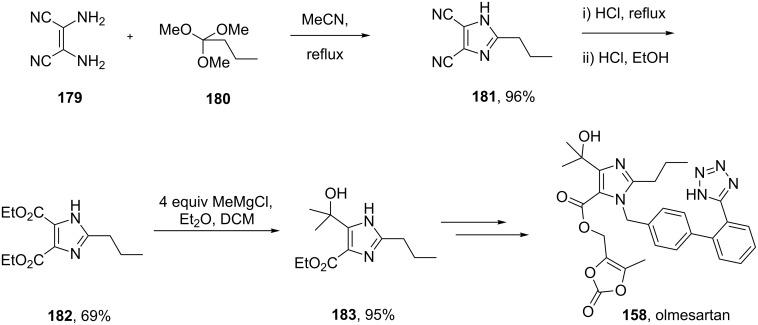
Preparation of the imidazole ring in olmesartan.

The structurally related imidazole core of olmesartan is formed in a different fashion (Scheme 36). Condensation between diaminomaleonitrile and trimethyl orthobutyrate furnishes the trisubstituted imidazole **181** in high yield [53–54]. Acid-mediated nitrile hydrolysis followed by esterification results in the corresponding diester unit **182**. Treatment of **182** with four equivalents of methylmagnesium chloride in a mixture of diethyl ether and dichloromethane selectively provides tertiary alcohol **183**. In subsequent steps this imidazole is alkylated with the tetrazole containing biphenyl appendage, followed by ester hydrolysis and alkylation of the resulting carboxylate with 4-(chloromethyl)-5-methyl-2-oxo-1,3-dioxole to yield olmesartan (Scheme 36).

**Scheme 37:**
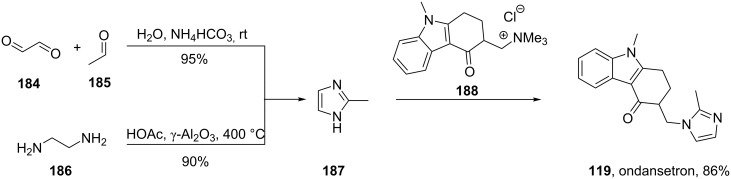
Synthesis of ondansetron.

The imidazole motif present in ondansetron (**119**), a prototypic 5-HT_3_ receptor antagonist, is incorporated into the molecule by substitution of a trimethylammonium functionality of an advanced intermediate **188** by 2-methylimidazole (**187**, Scheme 37) [55]. Alternatively, this substitution can be performed on a cyclohexenone derivative prior to the Fischer indole synthesis (Scheme 27) [38]. The required imidazole **187** itself can be prepared via a variety of methods. For example, a process involving acetaldehyde, glyoxal and ammonium carbonate furnishes the desired compound in an excellent 95% yield (Scheme 37). Also, a condensation reaction between ethylenediamine and acetic acid catalysed by γ-Al_2_O_3_ at high temperatures gives 2-methylimidazole (**187**) in approximately 90% yield [56].

**Scheme 38:**
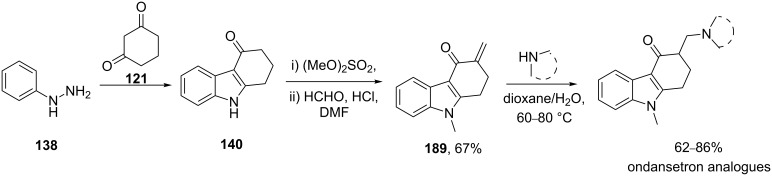
Alternative route to ondansetron and its analogues.

Elz and Heil [57] prepared the 1,2,3,9-tetrahydro-4*H*-carbazol-4-one (**140**) by the reaction of phenylhydrazine (**138**) with cyclohexan-1,3-dione (Scheme 38). Classical *N*-methylation with dimethyl sulfate followed by introduction of an exocyclic double bond using paraformaldehyde in DMF under acidic conditions furnishes the Michael acceptor **189**, which then undergoes conjugate addition with various amines (Scheme 38). This route has been used to prepare several ondansetron analogues based on different amine components.

### Benzimidazoles

**Scheme 39:**
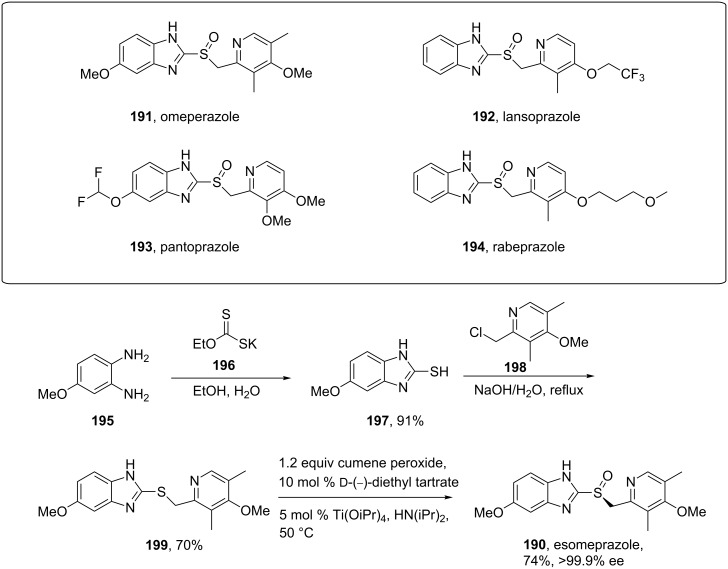
Proton pump inhibitors and synthesis of esomeprazole.

Nearly all benzimidazole containing drugs such as esomeprazole (**190**, Nexium), omeprazole (**191**, Prilosec), lansoprazole (**192**, Prevacid), pantoprazole (**193**, Protonix) and rabeprazole (**194**, Aciphex) are proton pump inhibitors. The common feature in their synthesis is the double condensation of a 1,2-diaminobenzene with potassium ethylxanthate (**196**) [58]. A typical synthesis of the methoxybenzimidazole system present in esomeprazole (omeprazole) and lansoprazole is shown in Scheme 39. For esomeprazole the subsequent steps involve an *S*-alkylation as well as an asymmetric oxidation of the newly formed thioether [59–60].

**Scheme 40:**
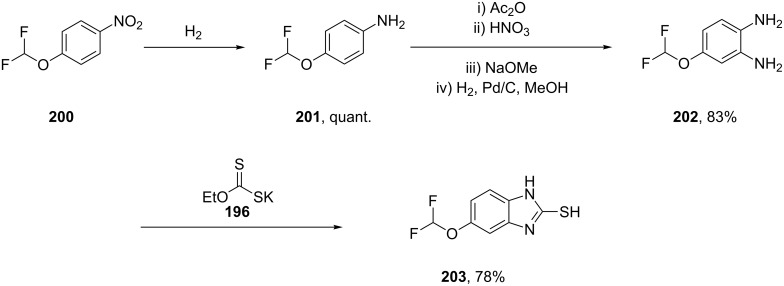
Synthesis of benzimidazole core pantoprazole.

Additional structural diversity in the aniline component can be introduced by protection, nitration, deprotection and reduction of the starting amine compound **201**. Scheme 40 for instance shows this in the synthesis of the benzimidazole core of pantoprazole [61].

**Figure 6:**
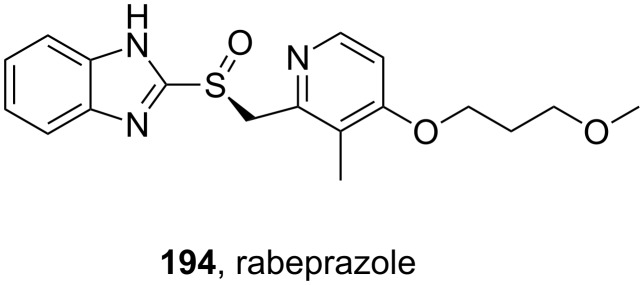
Structure of rabeprazole **194**.

The benzimidazole ring in rabeprazole (**194**, Figure 6) is only substituted at position 2 and can be easily prepared by the same procedure.

**Scheme 41:**
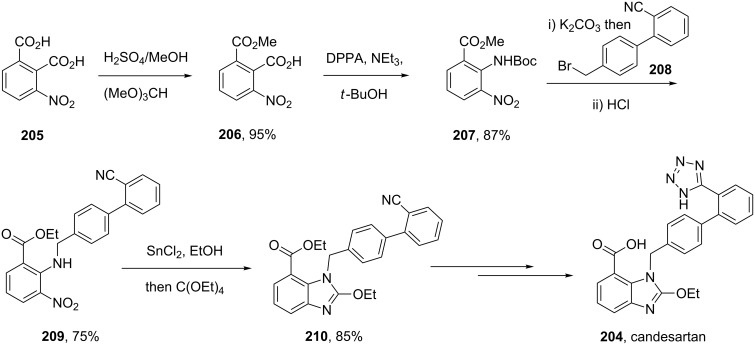
Synthesis of candesartan.

**Scheme 42:**
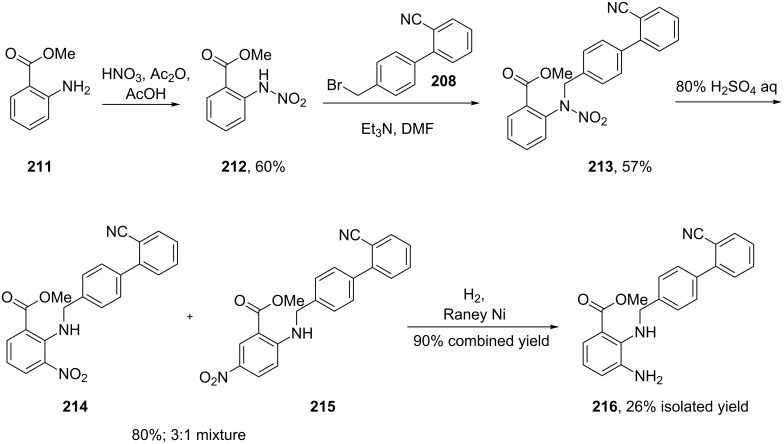
Alternative access to the candesartan key intermediate **216**.

In a similar strategy, the benzimidazole structure **210** in the angiotensin II antagonist candesartan (**204**, Atacand) is formed from an advanced diaminobenzene derivative, which in turn is prepared by a tin-mediated nitro reduction to form a highly substituted diaminobenzene (Scheme 41). This general strategy involving initial alkylation of **207** is often employed to control the regioselectivity of the imidazole formation. The benzimidazole ring is then assembled by treating this diamine with tetraethyl orthocarbonate under Lewis acid conditions. The synthesis is concluded by installation of the tetrazole ring and acetal side chain, the latter is cleaved under physiological conditions, making candesartan a pro-drug in the same way as olmesartan [62]. This synthesis of the benzimidazole precursor makes use of a 1,2,3-trisubstituted nitrophthalic acid **205**, which had to be selectively monoesterified and subjected to a Curtius rearrangement, which is cumbersome when the reaction is scaled up. Alternative routes to such trisubstituted benzenes include directed *ortho*-lithiation of 1,3-disubstituted benzenes followed by trapping of the anion with a nitrogen electrophile [63]. As a result a new route to access the key building block has been proposed which makes use of a less widely utilised acid catalysed rearrangement of methyl *N*-nitroanthranilate (**212**) which is obtained by *N*-nitration of methyl anthranilate (**211**, Scheme 42). Alkylation of the intermediate with the biphenylmethyl bromide **208** under mildly basic conditions yields adduct **213**. Upon treatment of this compound with 80% aqueous sulfuric acid a rearrangement to furnish a mixture of the 3- and 5-nitro derivatives occurs, which unfortunately at this stage, could not be separated by crystallisation. However, when this mixture was subjected to catalytic hydrogenation with Raney nickel a separable mixture of the corresponding diaminobenzoates was obtained (Scheme 42) [64].

**Scheme 43:**
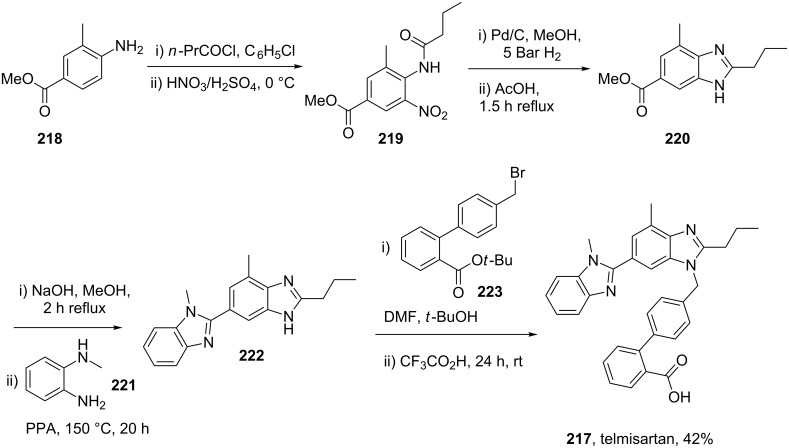
.Medicinal chemistry route to telmisartan.

Telmisartan (**217**, Micardis) is a well known angiotensin II receptor antagonist used in the treatment of hypertension and, heart and bladder diseases. Its pharmacophore consists of two linked benzimidazoles and a biphenyl unit (Scheme 43). As shown previously, such benzimidazoles can be formed through the condensation reaction of a 1,2-diaminobenzene and a suitable functionalised carbonyl compound. However, in the case of telmisartan other inherent functionalities such as an ester are present which leads to the formation of several by-products and consequently significantly lower yields. Another obvious drawback of the initially described route [65] when considering scale up, is the number of sequential steps in the synthesis (8 steps, 21% overall yield) (Scheme 43).

**Scheme 44:**
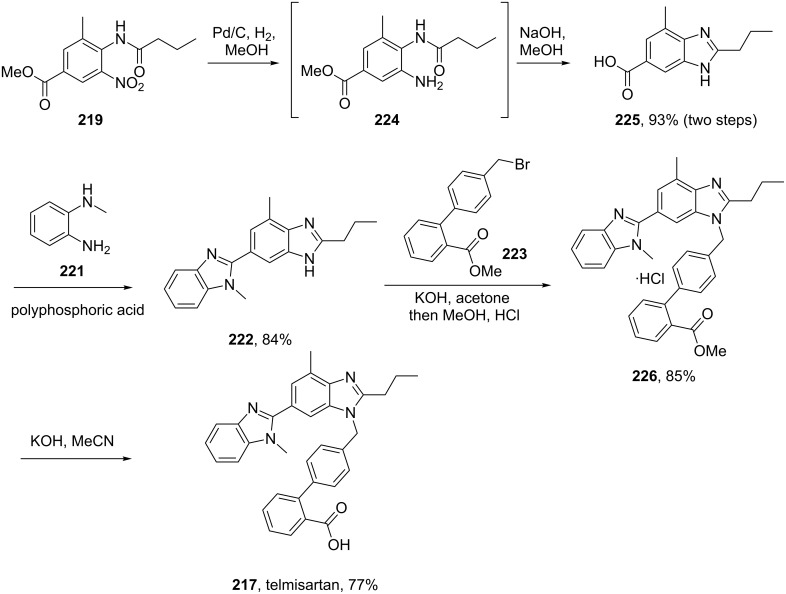
Improved synthesis of telmisartan.

Hence an improved synthesis was sought [66]. This revised route utilised a palladium-mediated reduction of the highly substituted nitrobenzene derivative **219** to afford aniline **224** which under basic conditions ring closes to the corresponding benzimidazole **225**. Under the same reaction conditions, the hydrolysis of the methyl ester also occurs which allows the introduction of the second imidazole group in the subsequent condensation step. Sterically directed alkylation of the free imidazole nitrogen with the required biphenyl derivative **223** in basic media yields the methyl ester **226** which is isolated, after work-up and solvent removal, as the hydrochloride salt in 85% yield. HPLC analysis showed a purity of >99.5% which is by far superior to the previously reported synthesis [65]. Finally, hydrolysis of the methyl ester provides telmisartan in an overall yield of about 50% compared to 21% as previously obtained (Scheme 44).

### Imidazopyridine

**Scheme 45:**
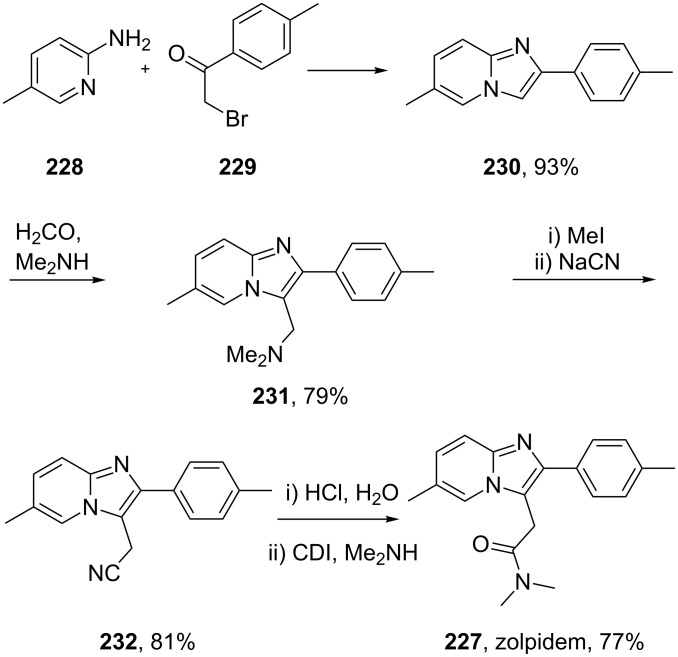
Synthesis of zolpidem.

Zolpidem (**227**) is a non-benzodiazepine hypnotic, and is part of the imidazopyridine class of pharmaceuticals. It is an agonist for the target receptor of γ-aminobutyric acid (GABA), an inhibitory neurotransmitter. Zolpidem binds to GABA receptors at the same site as typical benzodiazepines. This drug is preferred to benzodiazepines for long term use since benzodiazepines lead to a higher tolerance as well as physical dependence. The standard route to this scaffold is the cyclocondensation of a functional 2-aminopyridine with an α-bromo-carbonyl compound [67–68]. In the case of zolpidem, the amide moiety in the 3-position of the ring system is introduced via a Friedel–Crafts/Mannich-type alkylation starting either from formaldehyde and dimethylamine or 2,2-dimethoxy-*N*,*N*-dimethylacetamide (Scheme 45).

**Scheme 46:**
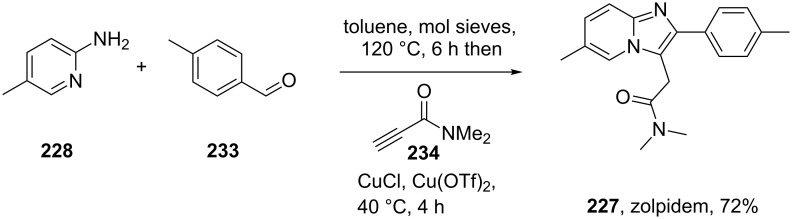
Copper-catalysed 3-component coupling towards zolpidem.

In a recent study a more straightforward and general copper-catalysed three component coupling leading to imidazopyridines has been reported [69]. For this reaction 2-amino-5-methylpyridine (**228**) was condensed with an aldehyde to form an intermediate imine to which is added a terminal alkyne in the presence of copper(I) chloride. A copper(II) triflate catalyst is then used to promote a Lewis acid promoted 5-*exo*-*dig* heteroannulation to furnish, after chromatography, the bicyclic structure in good overall isolated yield (Scheme 46).

Despite this synthesis being much shorter and convergent it has some limitations since the entire procedure needs to be performed in a glove box and has consequently only been reported on small scale.

### Pyrazole

**Figure 7:**
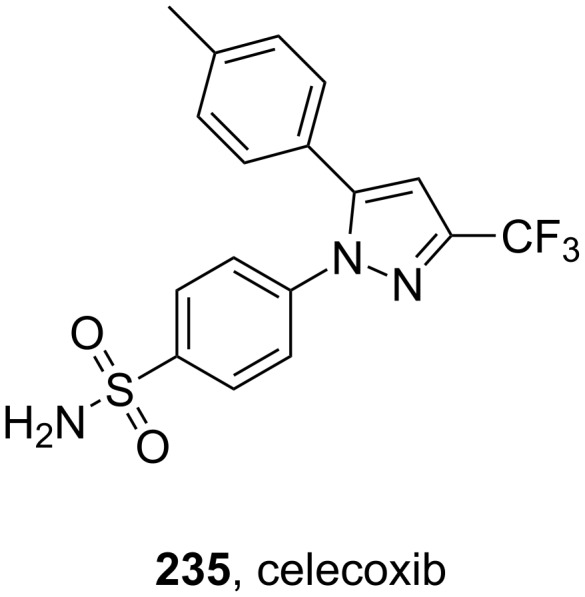
Structure of celecoxib.

Selective inhibitors of cyclooxygenase-2 (COX-2) are widely used for their anti-inflammatory effects and have shown less gastrointestinal side effects when compared to other anti-inflammatory agents, notably non-steroidal anti-inflammatory drugs (NSAIDs) such as aspirin and ibuprofen, which inhibit both COX-1 and COX-2. The central ring is usually a five-membered aromatic system, which is diaryl-substituted with a (*Z*)-stilbene-like linking structure. A polar sulfonamide group or biologically equivalent unit is usually present at the *para*-position of one of the aryl rings and is believed to promote binding to a hydrophilic pocket close to the active site of COX-2. Celecoxib (**235**, Celebrex, Figure 7) belongs to the group of selective COX-2 inhibitors acting on the prostaglandin G/H synthase 2 as well as 3-phosphoinositide-dependent protein kinase 1 and is marketed by Pfizer. Interestingly, celecoxib has also been approved for familial adenomatous polyposis demonstrating its ability to induce apoptosis in certain cancer cell lines [70]. As this activity is not shared with all COX-2 inhibitors, it is believed that the structural features such as the polar sulfonamide group, the lipophilic tolyl moiety and the trifluoromethylated pyrazole core with its negative electrostatic potential play a key role in apoptosis induction. Consequently, the anti-inflammatory and apoptosis inducing properties of celecoxib are assumed to result via different modes of action.

In a recent study [71], celecoxib has also been shown to be a rapid, freely reversible, competitive inhibitor of COX-1. This result was supported by X-ray crystallographic evidence, where celecoxib was shown to bind to one subunit of the COX-1-dimer, implying that drugs like aspirin then bind to the other monomer of the same enzyme consequently slowing down the irreversible acetylation of a serine residue by aspirin itself. This finding is relevant as aspirin is clinically used in combination with celecoxib to attenuate its cardiovascular side effects. Based on this in vitro study, it is suggested that the cardioprotective effects of low-dose aspirin on COX-1 might be reduced when administered with celecoxib. Further studies are currently underway to elucidate the full sympathetic action of co-administration.

**Scheme 47:**
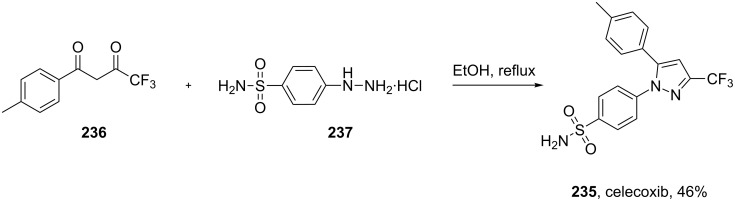
Preparation of celecoxib.

The common synthetic route to the diarylpyrazole ring of celecoxib (**235**) is a direct condensation of the 1,3-dicarbonyl compound **236** and the substituted hydrazine **237** [72–73] (Scheme 47).

**Scheme 48:**
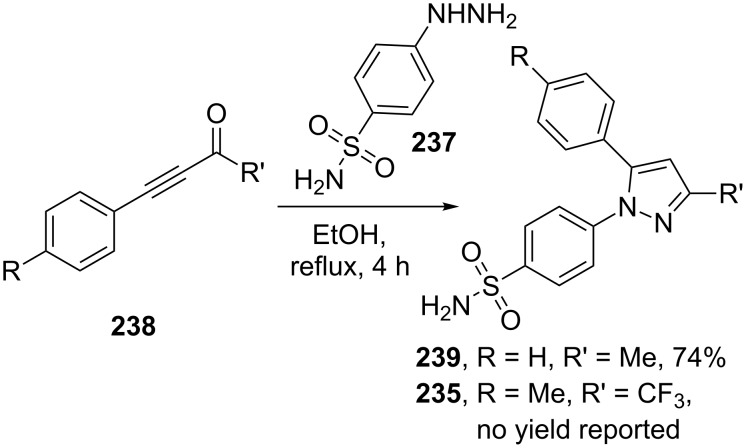
Alternative synthesis of celecoxib.

The immediate downside to this approach is the generation of regioisomeric mixtures. However, this is often of minor concern. In the commercial process only 2–5% of the unwanted regioisomer is produced, with the purified pyrazole being obtained by crystallisation. It has been claimed that minimising the diketone’s exposure to water prior to its reaction with the hydrazine significantly reduces the formation of the unwanted regioisomer by avoiding the formation of a hydrate at the carbonyl bearing the more electronegative CF_3_-group. Alternatively, in order to circumvent the regioselectivity issue, other pyrazole syntheses have been used. For example, the substituted aryl hydrazine **237** can be reacted with trifluoromethyl butynone in a one pot reaction. A Michael addition/cyclisation sequence renders only the desired regioisomer of the pyrazole [74]. However, preparation of the alkyne intermediates involves a number of steps and often requires extensive column chromatography, which makes it an unattractive method for synthesis on a larger scale (Scheme 48).

**Scheme 49:**
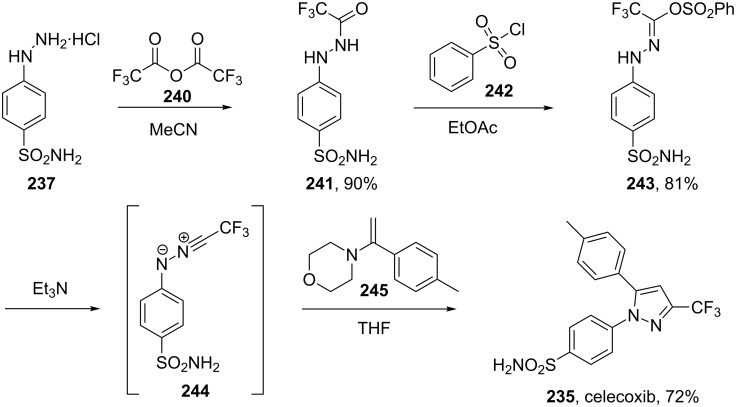
Regioselective access to celecoxib.

A novel 1,3-dipolar cycloaddition between a nitrile imide **244** and an appropriately substituted olefin **245** has also been used to obtain the corresponding trisubstituted pyrazole [75]. The final synthesis and its dipole precursor are represented in Scheme 49.

Since this is a LUMO-dipole/HOMO-dipolarophile controlled reaction, an electron-rich alkene is required. Therefore, when the morpholine derived enamine **245** was used the desired 1,3,5-substituted pyrazole was formed with 100% regioselectivity (Scheme 49). This high regioselectivity is only obtained when a 1,1-disubstituted enamine is used, the corresponding 1,2-disubstituted enamine yields mainly the other regioisomer.

### Indazole

**Scheme 50:**
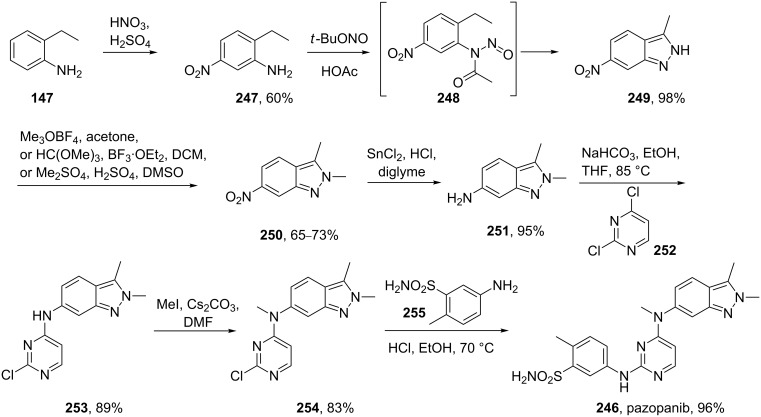
Synthesis of pazopanib.

Pazopanib (**246**, Votrient) is a new potent multi-target tyrosine kinase inhibitor for various human cancer cell lines. Pazopanib is considered a promising replacement treatment to imatinib and sunitinib and was approved for renal cell carcinoma by the FDA in late 2009. The indazole system is built up via diazotisation and spontaneous cyclisation of 2-ethyl-5-nitroaniline (**247**) using *tert*-butyl nitrite. The resulting indazole structure **249** can be methylated entirely regioselectively with either Meerwein’s salt, trimethyl orthoformate or dimethyl sulfate. A tin-mediated reduction of the nitro group unmasks the aniline which undergoes nucleophilic aromatic substitution to introduce the pyrimidine system with the formation of **253**. Methylation of the secondary amine function with methyl iodide prior to a second S_N_Ar reaction with a sulfonamide-derived aniline affords pazopanib (Scheme 50) [76–77].

### 1,2,4-Triazole

**Scheme 51:**
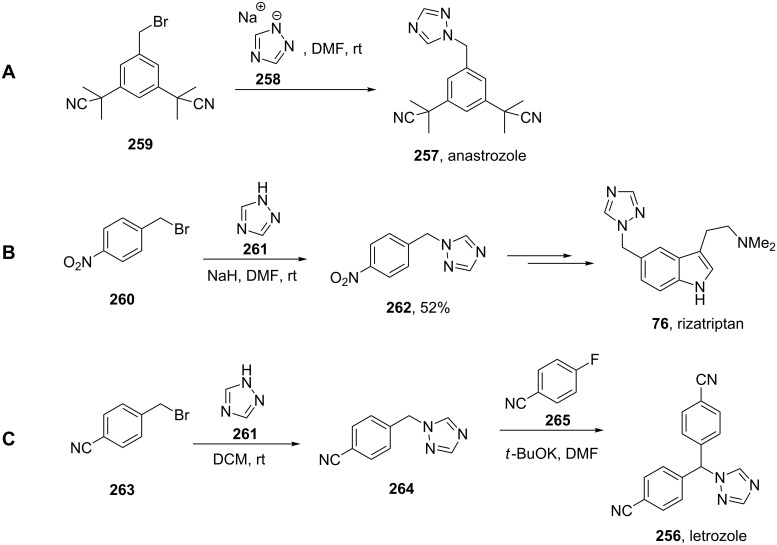
Syntheses of anastrozole, rizatriptan and letrozole.

Although many common antifungal drugs contain at least one 1,2,4-triazole ring, barely any of these drugs are represented in the top 200 drugs based on the value of sales. This observation obviously reflects more the price differential of the drug class rather than the utility of the heterocycle. In general, the triazole containing drugs belong to two groups of therapeutic agents: selective and non-steroidal aromatase inhibitors which are used in the treatment of early and advanced breast cancer in postmenopausal women, e.g., letrozole (**256**, Femara) and anastrozole (**257**, Arimidex) and 5-HT_1_ agonist triptan drugs such as rizatriptan (**76**, Maxalt) which are prescribed for migraine headaches. The large scale syntheses of all these compounds use the commercially available 1,2,4-triazolyl sodium salt **258** in an alkylation reaction with the corresponding benzyl bromide derivative as the key step (Scheme 51) [78].

**Scheme 52:**
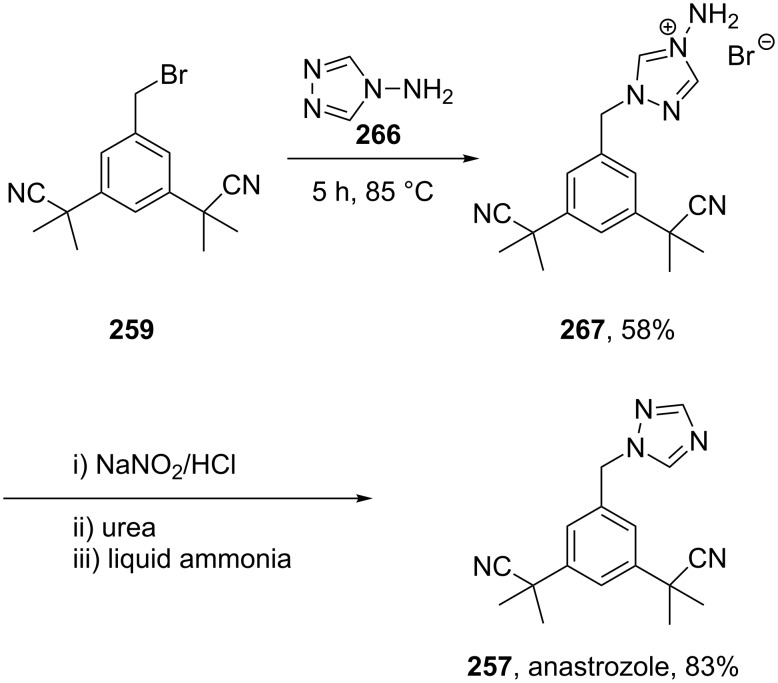
Regioselective synthesis of anastrozole.

One major problem with this route towards anastrozole (**257**) (Scheme 51) is the formation of the non-desired regioisomer, which is often produced in between 10–20% and must be removed by means of crystallisation which results in a major loss of material [79]. In order to circumvent the formation of the undesired regioisomer, a strategy that is often used is to react the corresponding benzyl bromide component first with 4-amino-1,2,4-triazole (**266**) to form a quaternary ammonium salt. The latter can be deaminated to give anastrozole with no isomeric impurities (Scheme 52) [79].

**Scheme 53:**
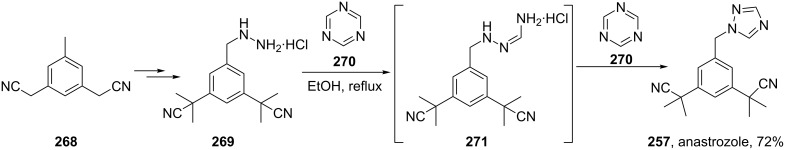
Triazine-mediated triazole formation towards anastrozole.

Other synthetic routes to 1,2,4-triazoles have also been reported. For example, the desired triazole ring of anastrozole can be obtained by reacting an appropriately substituted hydrazine hydrochloride salt **269** with triazine (**270**) (Scheme 53) [80].

It has been proposed that this novel transformation occurs by a two step process: Initially, a molecule of triazine undergoes condensation and ring cleavage with the hydrazine to generate formamidrazone **271** which then immediately reacts with a second molecule of triazine to yield the 1,2,4-triazole [81]. Hence, in this case the triazine can be considered as a formamide donor.

**Scheme 54:**
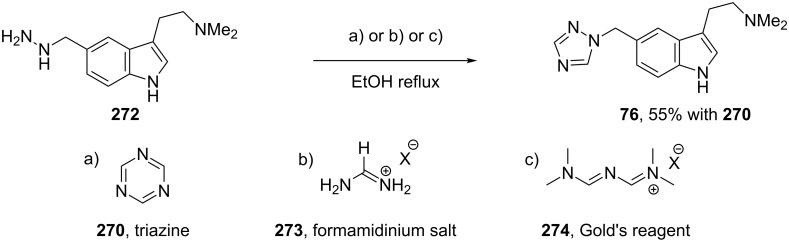
Alternative routes to 1,2,4-triazoles.

Rizatriptan (**76**) has also been prepared by both the above mentioned procedures, i.e., via the pre-made triazole or by treatment of a hydrazine hydrochloride salt with triazine (Scheme 54) [82]. Indeed, the latter protocol has been further expanded to make use of additional formamide surrogates such as a formamidinium salt **273** or Gold’s reagent (**274**) (Scheme 54).

**Scheme 55:**
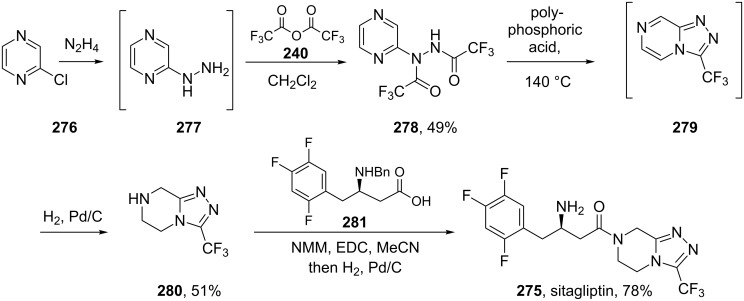
Initial synthetic route to sitagliptin.

**Figure 8:**
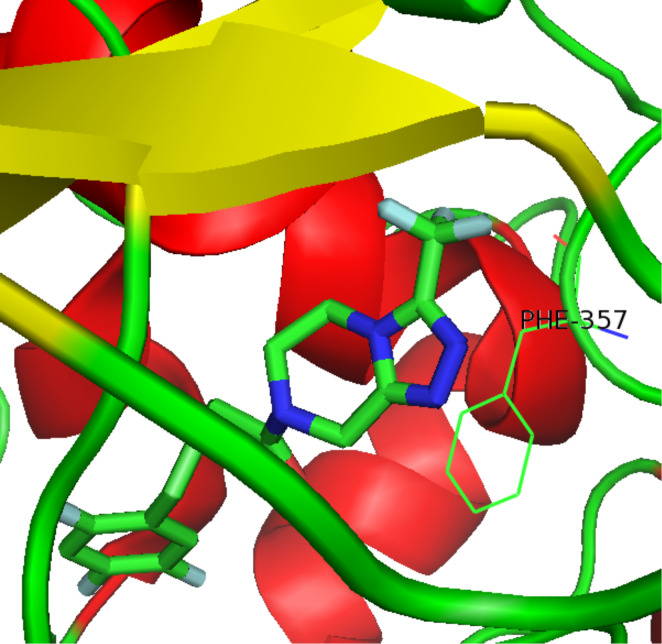
Binding of sitagliptin within DPP-IV.

Sitagliptin (**275**, Januvia) is a recently developed oral anti-diabetic drug which belongs to the dipeptyl peptidase DPP-IV inhibitor class. The inhibition of DPP-IV leads to increased incretin levels and the inhibition of glucagon release. Consequently, insulin secretion increases leading to decreased blood glucose levels in diabetes type II patients. Structurally, this novel antihyperglycaemic consists of a fluorinated β-amino acid which is coupled to a trifluoromethylated triazolopiperazine (Scheme 55). In SAR studies this fused heterocycle was found to be more metabolically stable compared to earlier leads that contained a simple piperazine ring. Furthermore, the triazolopiperazine is not only involved in a tight H-bond network within the active site of DPP-IV, but also in π-stacking with the aromatic ring of a nearby phenylalanine residue, whilst the trifluoromethyl group interacts with serine and arginine residues in a lipophilic pocket (Figure 8) [83].

In the discovery chemistry route [84] the heterocycle core was prepared from a S_N_Ar reaction between chloropyrazine (**276**) and excess hydrazine. Subsequent treatment of the substituted intermediate with trifluoroacetic anhydride furnished the corresponding bis-hydrazide **278** which underwent cyclisation at elevated temperatures in the presence of polyphosphoric acid. Finally, the partial hydrogenation of triazolopyrazine derivative **279** with palladium on carbon gave the core triazolopiperazine **280**. This was then coupled with carboxylic acid unit **281** under standard peptide bond forming conditions and subjected to palladium catalysed debenzylation to liberate the free amine functionality of sitagliptin (**275**) (Scheme 55).

**Scheme 56:**
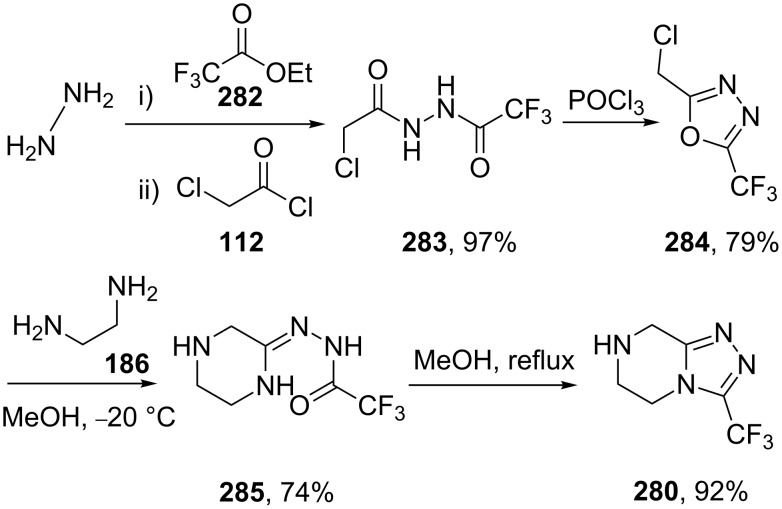
The process route to sitagliptin key intermediate **280**.

An inherent problem with this synthesis was the necessity for excess hydrazine in the first step of the sequence in addition to a requirement for an expensive and only moderately efficient palladium reduction in the penultimate step. Furthermore, the chloropyrazine starting material also proved to be unstable under a series of reaction conditions and gave rise to numerous by-products. An improved route was developed for the compounds large scale manufacture. The refined process route (Scheme 56) starts with a sequential bis-acylation of hydrazine with ethyl trifluoroacetate and chloroacetyl chloride [85]. The resulting hydrazide **283** was then subjected to cyclodehydration using phosphoryl chloride to give a chloromethyl oxadiazole derivative **284**. In a cleverly staged transformation, this compound was treated with diaminoethane to yield the piperazine ring **285**, which, on heating under reflux in methanol, undergoes a further condensation with the attached hydrazide to furnish the desired triazolopiperazine ring **280** directly.

**Scheme 57:**
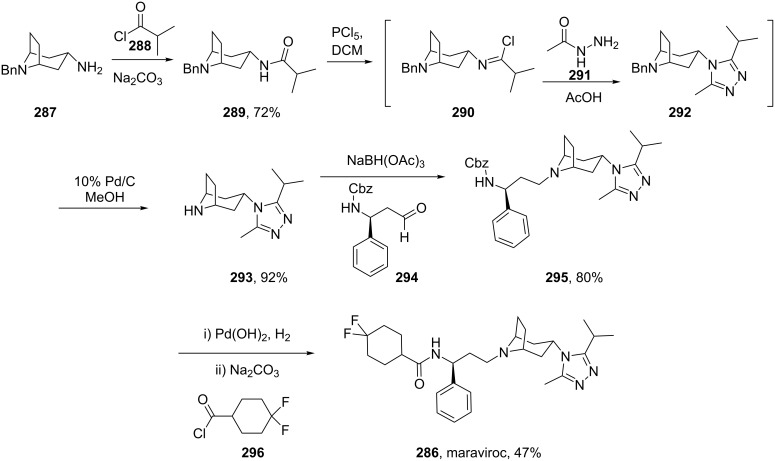
Synthesis of maraviroc.

A novel and very promising HIV treatment is Pfizer’s maraviroc (**286**, Celsentri). HIV uses a member of the G-protein coupled receptor family called CCR-5 as an anchor to attach itself to white blood cells such as T-cells and macrophages followed by viral fusion and entry into white blood cells. Maraviroc blocks this pathway by acting as an antagonist for the CCR-5 receptor hence disrupting HIV life cycle. The structural features of this molecule are a geminal difluorocyclohexyl carboxamide which is linked to a β-aminoacid, and a tropinone-type unit bound to a 1,2,4-triazole ring. Relatively simple and straightforward chemical transformations are used to assemble the main fragments of maraviroc such as amide bond formation and reductive amination (Scheme 57) [86]. The triazole ring incorporation is achieved at an early stage by *N*-acylation of the tropinone fragment **287** with 2-methylpropanoyl chloride (**288**). The resulting amide **289** is then converted to the corresponding imidoyl chloride **290** using phosphorous pentachloride in dichloromethane (which proved to be superior to phosphoryl chloride) followed by condensation with acetic hydrazide (**291**). It was found that the dryness of the acetic hydrazide was crucial in order to minimise the hydrolysis of the starting amide **289**.

**Scheme 58:**
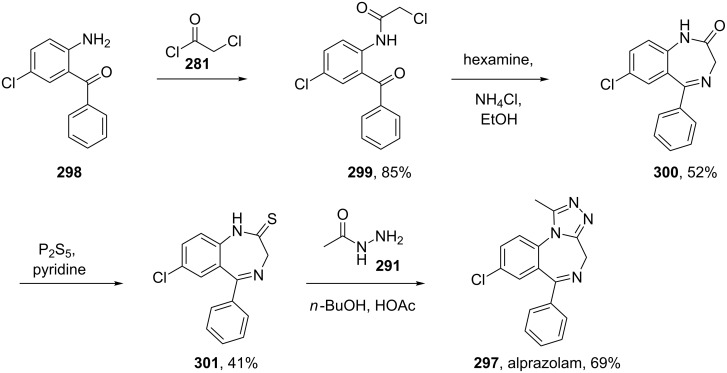
Synthesis of alprazolam.

Benzodiazepines are a well known class of compounds with a wide range of CNS-related activities. Moreover, the attachment of a third ring has been found to impact greatly on the pharmacological profile of these drugs. A representative of this compound class is alprazolam (**297**, Xanax) which contains a 1,2,4-triazole fused to the benzodiazepine core. The synthesis of this molecule [87–88] (Scheme 58) can be accomplished in a short sequence of steps starting by acylation of 2-amino-5-chlorobenzophenone (**298**) with chloroacetyl chloride (**281**) to give the amide derivative **299**. The latter undergoes an interesting ring closure reaction in the presence of hexamine and ammonium chloride and the resulting seven membered lactam **300** can then be converted into its thioamide analogue **301** with P_2_S_5_ in pyridine. Finally, the reaction of **301** with acetyl hydrazide (**291**) catalysed by acetic acid furnishes the triazole ring fused to the benzodiazepine core.

**Scheme 59:**
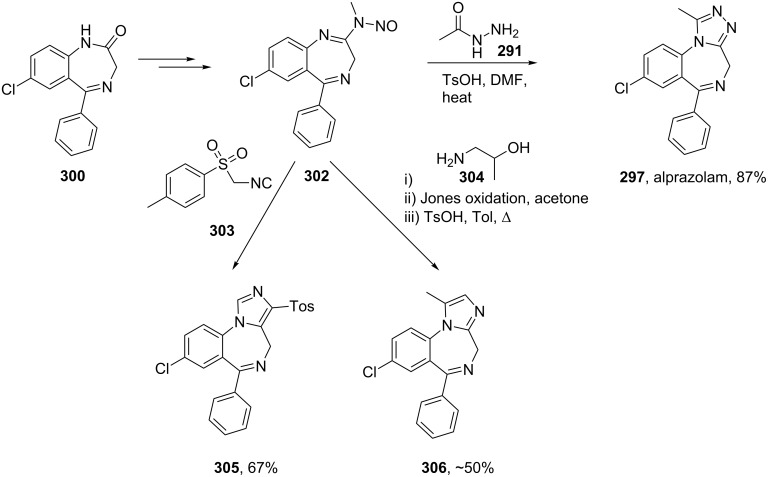
The use of *N*-nitrosoamidine derivatives in the preparation of fused benzodiazepines.

Another approach [89] makes use of 1,4-benzodiazepine-*N*-nitrosamidine **302** as the starting material which when treated with acetyl hydrazide (**291**) undergoes the final ring closure. This procedure can also be employed to prepare the related series of imidazobenzodiazepines if TosMIC (**303**) or the aminopropanol **304** are used as nucleophiles (Scheme 59).

**Figure 9:**
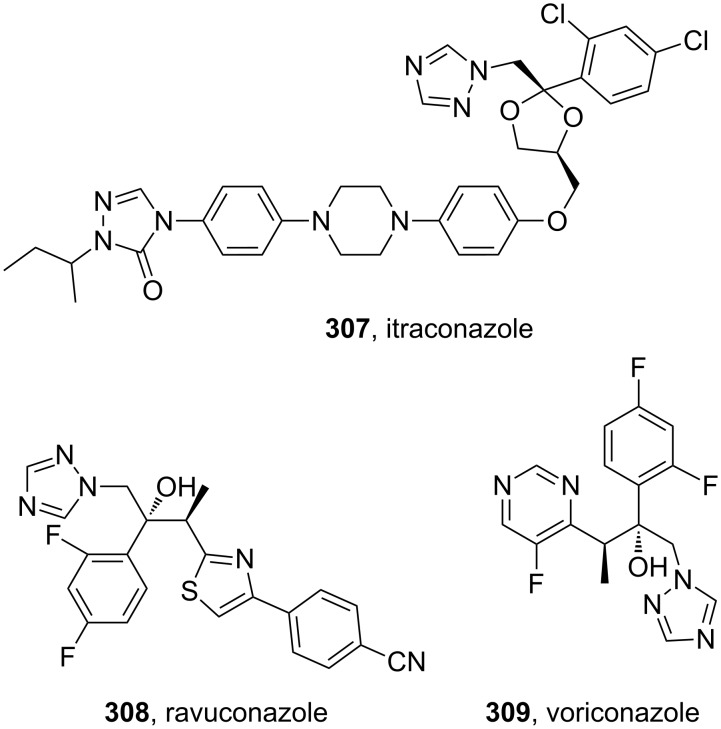
Structures of itraconazole, ravuconazole and voriconazole.

Triazole containing antifungal compounds (Figure 9) are a well known group of pharmaceuticals but only a few members are represented in the list of best selling drugs, e.g., itraconazole (**307**, Sporanox), ravuconazole (**308**, BMS-207147) and voriconazole (**309**, Vfend).

**Scheme 60:**
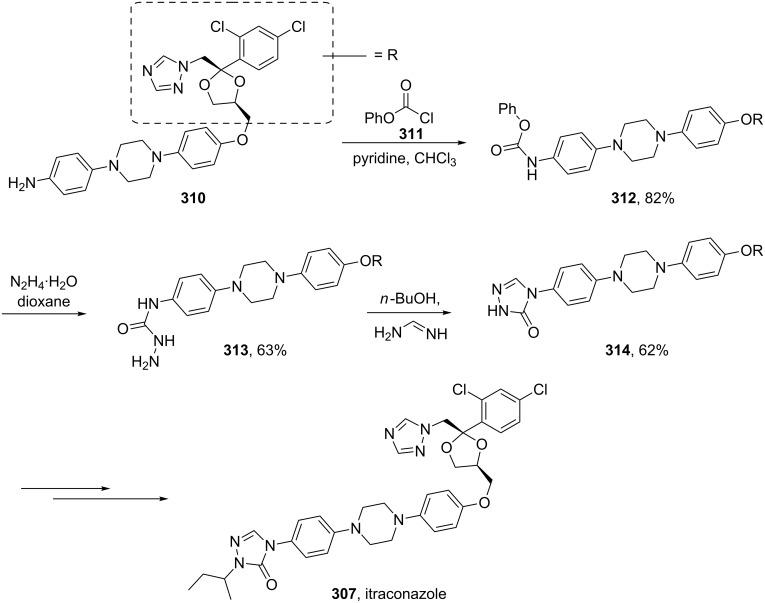
Synthesis of itraconazole.

All members of this class share a common biological activity being inhibitors of fungal cytochrome P-450 oxidase-mediated synthesis of ergosterol. Itraconazole, marketed by Janssen Pharmaceuticals, consists of two triazole subunits as well as a central diarylpiperazine unit (Figure 9). In one synthesis of itraconazole [90] 1,2,4-triazole was introduced via direct nucleophilic substitution. However, due to the previously discussed regioselectivity issues, extensive chromatographic purification was required following this step. In the latter stages of the synthesis, the elaborated aniline derivative **310** was trapped with phenyl chloroformate to form the corresponding carbamate which was then converted into triazolone **314** by a double condensation reaction with hydrazine and formamidine. Finally, simple attachment of an isobutyl group completes the synthesis (Scheme 60).

### 1,2,3-Triazole

**Scheme 61:**
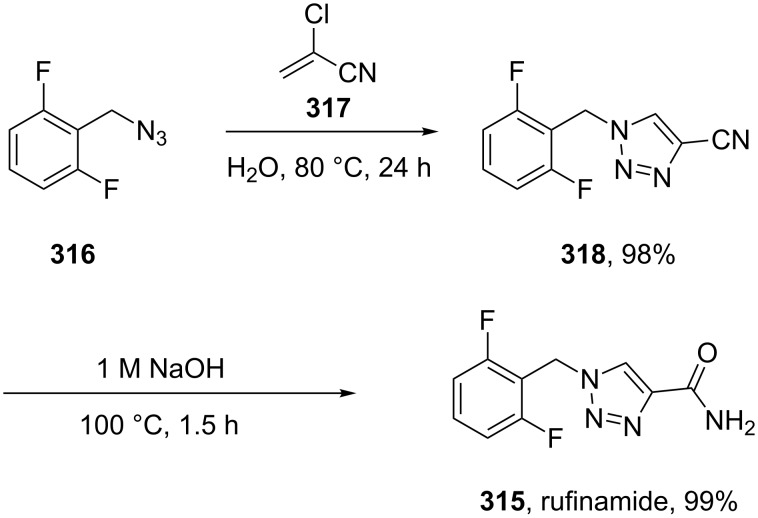
Synthesis of rufinamide.

Rufinamide (**315**, Inovelon) is Novartis’ new CNS-active compound used in the treatment of epilepsy. The common route [91–92] to the triazole ring present in this compound involves the reaction of 2,6-difluorobenzyl azide (**316**) with 2-chloro acrylonitrile **317** at temperatures around 80 °C in aqueous medium (Scheme 61). This transformation, which can be described either as a conjugate addition–elimination sequence or as a [3 + 2] cycloaddition followed by elimination was found to work best in a biphasic system, where the resulting HCl was retained in the aqueous phase thereby reducing overall amounts of polymerisation of the 2-chloroacrylonitrile starting material. In the final step the nitrile group is quantitatively hydrolysed under basic conditions to the primary amide.

Apart from this patented route, an improved approach has recently been described [93]. In this work it was shown that the highly toxic and flammable 2-chloroacrylonitrile can be readily substituted with the less toxic and less expensive methyl 3-methoxyacrylate. After thermal cycloaddition, the methyl ester is converted to the corresponding amide by the addition of methanolic ammonia. Overall, this process can be performed as a single pot procedure on a multi-gram scale to afford rufinamide in a similarly high yield and generating less waste.

### Tetrazole

**Scheme 62:**
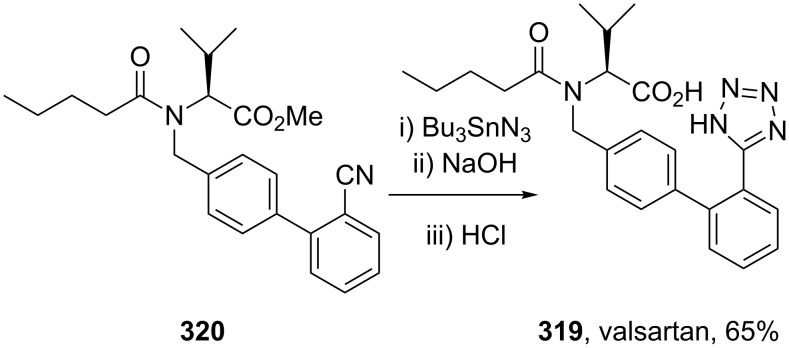
Representative tetrazole formation in valsartan.

The tetrazole motif as a bioisostere for a carboxyl group is a well documented structural replacement represented by five angiotensin II antagonists in the top selling 200 drugs. In order to generate the tetrazole ring, a nitrile is reacted with an azide, most commonly tributyltin azide. This is illustrated by the Novartis/Ciba-Geigy synthesis of valsartan (**319**, Diovan), where the tetrazole ring is constructed in the last step of the sequence (Scheme 62) [49].

**Figure 10:**
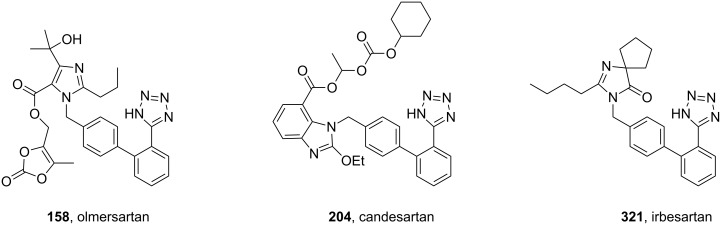
Structure of tetrazole containing olmesartan, candesartan and irbesartan.

The same approach is repeated in other angiotensin AT_2_ antagonists, such as olmesartan (**158**), candesartan (**204**) or irbesartan (**321**) (Figure 10).

**Scheme 63:**
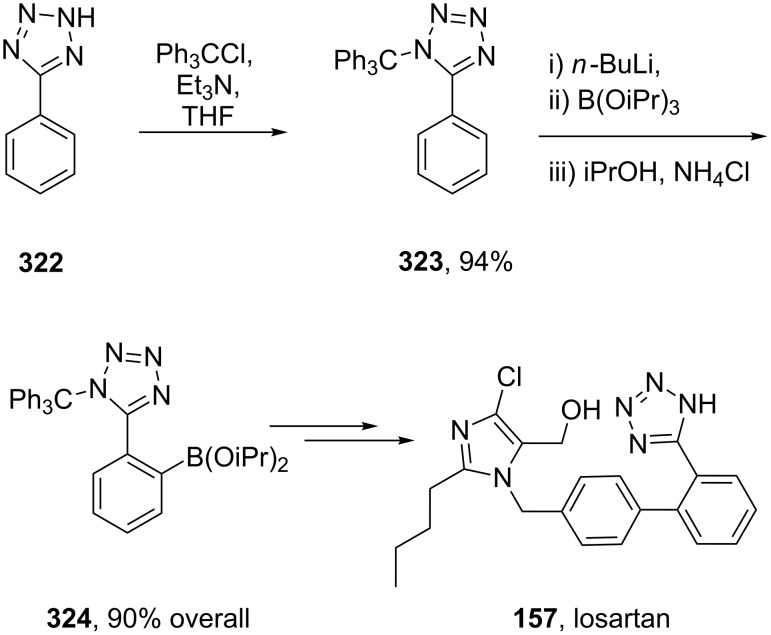
Early stage introduction of the tetrazole in losartan.

The tetrazole ring has also been introduced at the beginning of the synthesis, however, the heterocyclic ring, which has to be carried through all subsequent steps, often requires protection. One common protecting group is the trityl group as used in the synthesis of losartan (**157**) (Scheme 63) [51,94].

**Scheme 64:**
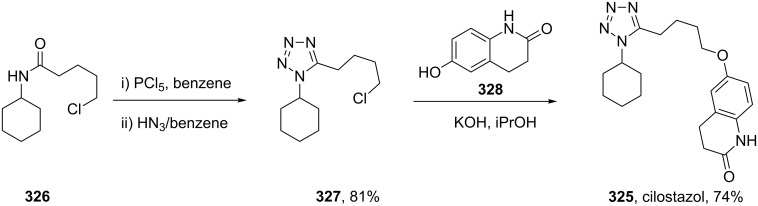
Synthesis of cilostazol.

The tetrazole ring also appears in cilostazol (**325**, Pletal) which is a selective PDE3 phosphodiesterase inhibitor used as a platelet aggregation inhibitor. The tetrazole ring **327** is prepared via the reaction of an in situ generated imidoyl chloride and hydrazoic acid delivered as a 10% solution in benzene [95–96]. Subsequent alkylation under Williamson conditions provides the final compound **325** in good yield (Scheme 64).

### Thiazole

**Figure 11:**
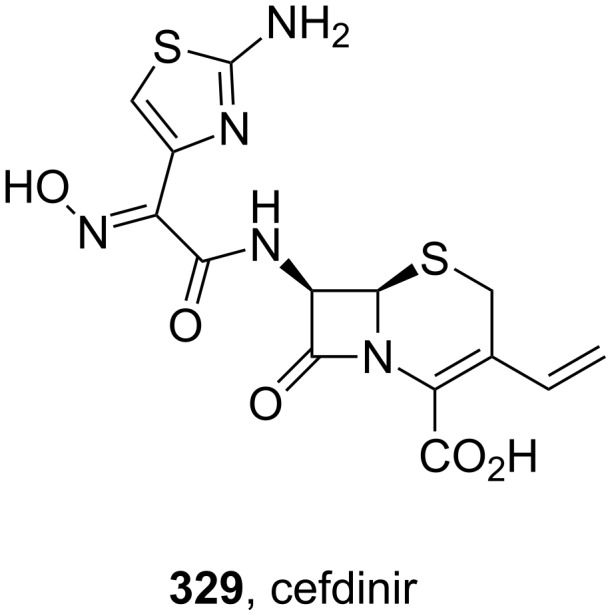
Structure of cefdinir.

The first example of a thiazole in the top 200 drugs listings is cefdinir (**329**, Omnicef, Figure 11), a semi-synthetic third generation cephalosporin which is administered orally and has an extended antibacterial activity against both gram-positive and gram-negative bacteria. The main feature of cefdinir is that it shows excellent activity against *Staphylococcus* species [97]. The thiazole ring in cefdinir shows that the heterocyclic structure in a drug not only affects its pharmacodynamic properties but can also influence its kinetics. It is believed that in the digestive tract iron(II) ions form chelate complexes with the thiazole ring and the oxime nitrogen atom and hence reduce the bioavailability of cefdinir [98].

**Scheme 65:**
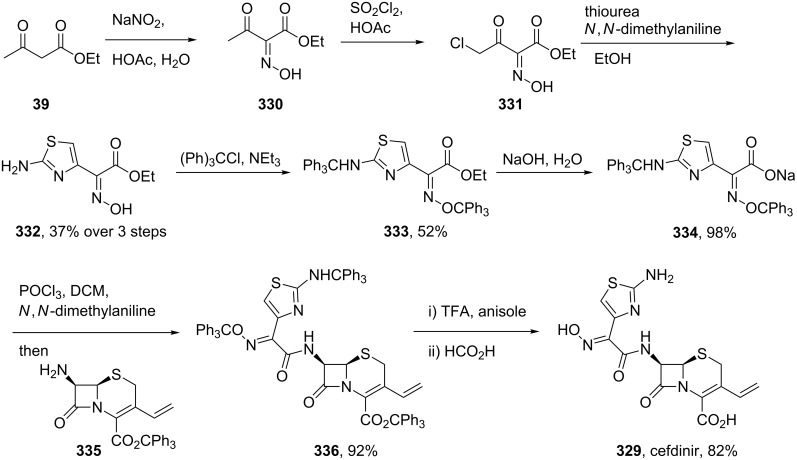
Semi-synthesis of cefdinir.

The convergent semi-synthesis of cefdinir can be achieved by coupling 7-amino-3-vinyl-3-cephem-4-carboxylic acid ester **335** with an advanced carboxylic acid derivative **334** which contains the elaborated thiazole motif (Scheme 65). This heterocyclic acid can itself be obtained from ethyl acetoacetate which is converted to an oxime **330** and α-chlorinated prior to a Hantzsch-type thiazole synthesis with thiourea in ethanol and *N*,*N*-dimethylaniline as the base [99]. Simultaneous trityl protection of the oxime and primary amine furnishes the desired coupling partner **334** in good overall yield. Hydroxide promoted ester hydrolysis was followed by treatment with phosphoryl chloride and the resulting acyl chloride coupled with the biologically derived lactam **335**. All three trityl protecting groups are simultaneously cleaved with TFA to furnish cefdinir **329**.

**Scheme 66:**
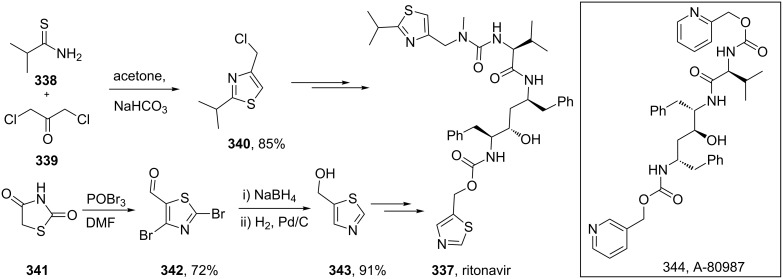
Thiazole syntheses towards ritonavir.

The HIV-1 protease inhibitor ritonavir (**337**, Norvir, Scheme 66) consists of two differently substituted thiazole rings, which are introduced at the later stages in the synthesis of this peptidomimetic antiviral compound. Interestingly, ritonavir itself is a result of further improvements on earlier candidates for the treatment of AIDS. A lead compound (**344**, A-80987) was also described by Abbott bearing pyridine rings on both ends of the peptidomimetic structure, which resulted in good bioavailability as required for orally administered drugs, but this compound had an insufficient plasma half-life. This was ascribed to the more electron-rich nature of the pyridine rings when compared to many other nitrogen containing heterocycles leading to a higher metabolic susceptibility. Consequently, the pyridine rings were replaced with less electron-rich thiazoles which resulted in both good bioavailability and a long plasma half-life. In addition, H-bonding of the 5-substituted thiazole to the backbone of Asp-30 of the HIV-1 protease is reported to be crucial with other substitution patterns showing reduced potency. The thiazole on the left-hand side is derived from the condensation between 2-methylpropane thioamide **338** and dichloroacetone **339** [100], whilst the other thiazole is obtained from the inexpensive 2,4-thiazolidinedione (**341**, Scheme 66) [101].

**Scheme 67:**
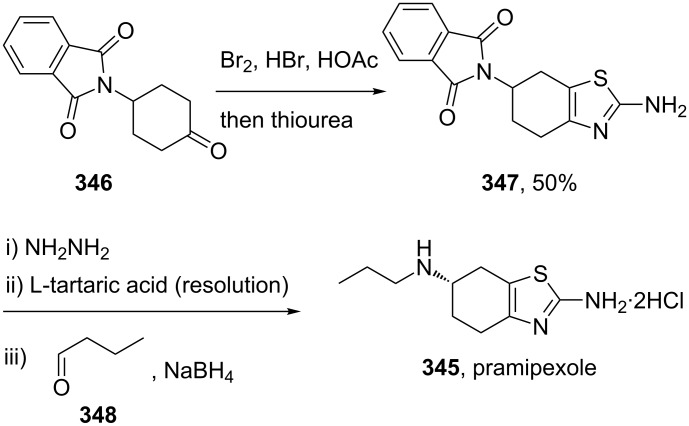
Synthesis towards pramipexole.

The dopamine D_2_-agonist pramipexole (**345**, Mirapex) consists of a fused bicyclic tetrahydrobenzothiazole motif, which is also prepared by a Hantzsch-type condensation between an α-brominated protected form of 4-aminocyclohexanone **346** and thiourea. Following deprotection, resolution with *L*-(+)-tartaric acid gives access to the *S*-enantiomer which undergoes reductive amination and can be isolated as the dihydrochloride salt (Scheme 67) [102].

**Scheme 68:**
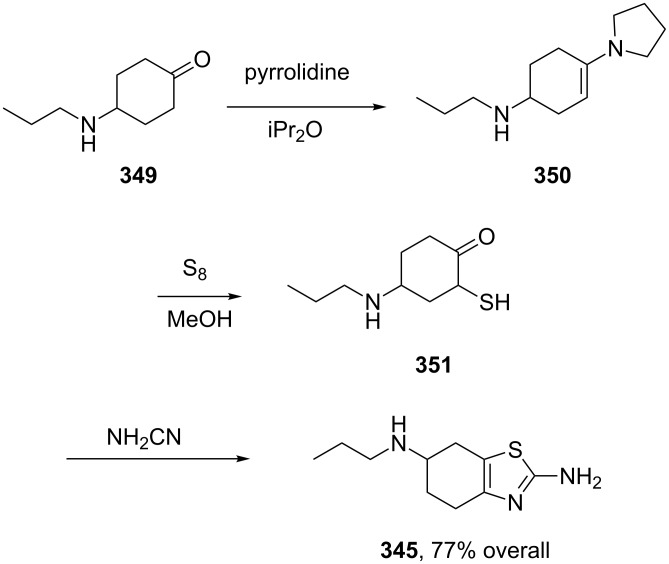
Alternative route to pramipexole.

Alternatively, the thiazole ring can be made in a one pot reaction from the corresponding enamine **350**, which is reacted with elemental sulfur to give the α-thioketone **351** (Scheme 68). This in turn can be treated with cyanamide to furnish the racemic thiazole **345** [103]. Resolution with (+)-ditoloyl-D-tartrate allows isolation of the desired *S*-enantiomer after treatment of the diastereomeric salt with sodium carbonate.

**Scheme 69:**
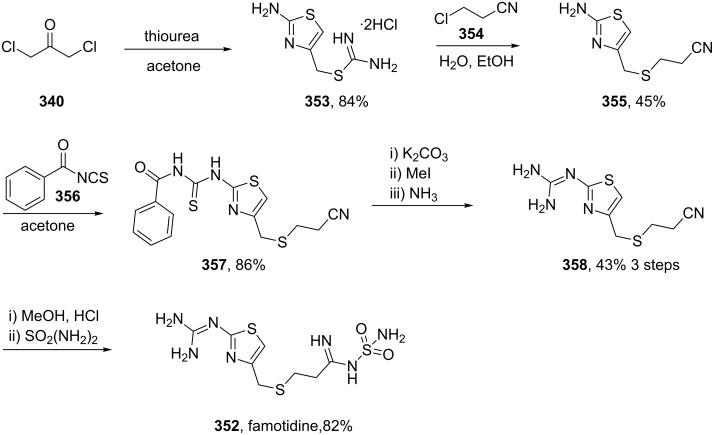
Synthesis of famotidine.

Famotidine (**352**, Pepcidine) is an H_2_-receptor antagonist similar to cimetidine which inhibits many isoenzymes of the hepatic CYP450 system and has the additional side effect of increasing the amount of gastric bacteria such as nitrate reducing bacteria. The structure of this ulcer therapeutic is very interesting and consists of a thiazole substituted guanidine and a sulfamoyl amidine. Although famotidine is orally administered, its solubility and hence bioavailability under acidic conditions, as found in the stomach, is relatively low. Recent reports have appeared that describe famotidine as a good ligand for various transition metals including copper and cobalt forming tetradentate {*N*,*N*,*S*,*N*}-coordination spheres as shown by single X-ray analysis [104]. It therefore seems feasible that certain common bioavailable cations might be involved in the absorption and activation of this thiazole containing compound. The synthesis of the thiazole ring [105–106] can be accomplished again by condensation of thiourea with dichloroacetone **340**. Alkylation of the isothiourea sulfur with 3-chloropropionitrile (**354**) and hydrolysis results in the formation of the substitution product **355**. Functionalisation of the resulting 2-amino group on the thiazole ring using benzoyl isothiocyanate generates the guanidine precursor **357**. A standard sequence of methylation and exchange with ammonia simultaneously cleaves the benzoyl group and unmasks the guanidine unit **358**. At the other peripheral of the molecule the nitrile functionality is converted to the imidate and subsequently coupled with sulfamide to yield famotidine (Scheme 69).

**Scheme 70:**
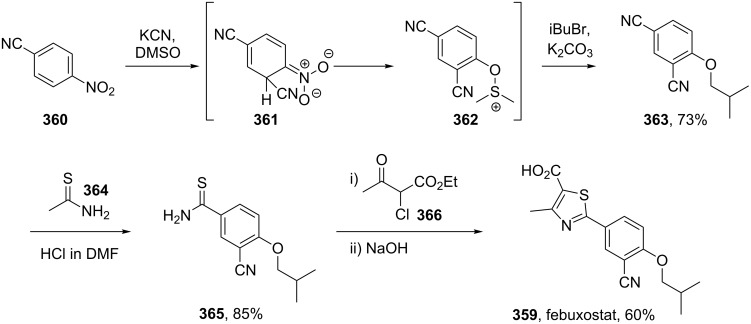
Efficient synthesis of the hyperuricemic febuxostat.

A final example of a thiazole containing drug is given in the novel xanthine oxidase inhibitor febuxostat (**359**, Uloric) which was approved by the FDA in 2009. This inhibitor works by blocking xanthine oxidase in a non-competitive fashion. Consequently, the amount of the oxidation product uric acid is reduced. Thus it is an efficient treatment for hyperuricemia in gout. In order to prepare febuxostat first a synthesis of the non-commercial 4-isobutoxy-1,3-dicyanobenzene building block (**363**), has to be conducted. An elegant way of achieving this was shown through the reaction of 4-nitrocyanobenzene (**360**) with potassium cyanide in dry DMSO followed by quenching with isobutyl bromide under basic conditions (Scheme 70). It is suggested that a Meisenheimer-complex intermediate **361** is initially formed, which after rearomatisation, undergoes nucleophilic aromatic substitution of the nitro group by the DMSO solvent [107]. Upon hydrolysis and *O*-alkylation the desired 4-isobutoxy-1,3-dicyanobenzene (**363**) is obtained in good overall yield. Subsequently, the less hindered nitrile is converted to the corresponding thioamide **365** in an intriguing reaction using thioacetamide (**364**). The thiazole ring is then formed by condensation with chloroacetoacetate **366** followed by ester hydrolysis (Scheme 70).

**Scheme 71:**
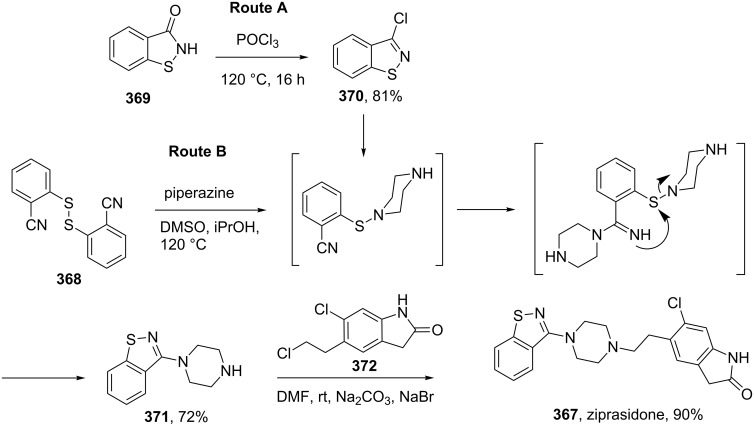
Synthesis of ziprasidone.

Ziprasidone (**367**, Geodon), is an atypical antipsychotic used to treat schizophrenia as well as mania and related bipolar disorder (Scheme 71). The exact pharmacological effect of ziprasidone is not simple to understand as it affects many subtypes of dopamine, adrenergic and serotonin receptors. However, like many antipsychotic drugs its main therapeutic activity is probably due to its antagonistic action on dopamine receptors. The molecule comprises of a 1,2-benzisothiazole core, which is readily prepared from commercially available benzo[*d*]isothiazol-3(2*H*)-one (**369**), a saccharin derivative [108]. The corresponding chloroimidate **370** formed by treatment of compound **369** with phosphoryl chloride is reacted with excess piperazine to afford intermediate **371** (Route A) [109]. Although this route enabled the synthesis of ziprasidone as its hydrochloride-salt, the nucleophilic aromatic substitution step (**370**→**371**) was poor and gave only 38% of the desired product [110]. Therefore an improved process was developed involving the oxidative coupling of piperazine with bis(2-cyanophenyl)disulfide (**368**) at elevated temperatures [109]. Due to the use of DMSO as a co-solvent and oxidant, each equivalent of the disulfide component was then converted into two equivalents of the product making the process more economically efficient. The final union was accomplished using standard Finkelstein alkylation conditions furnishing ziprasidone in 90% yield (Scheme 71) [111].

### Furan

**Figure 12:**
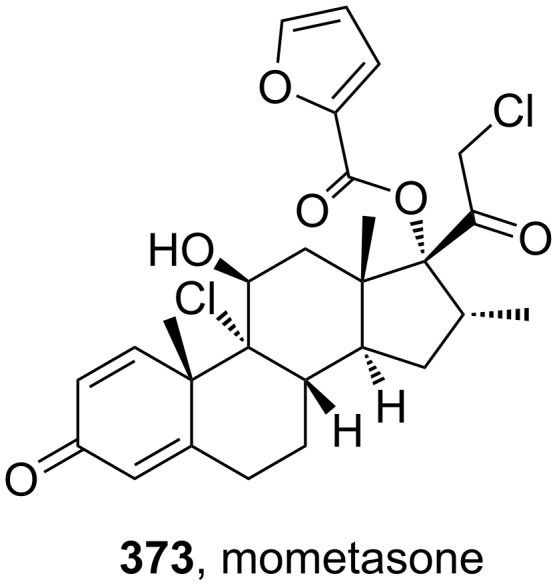
Structure of mometasone.

Mometasone (**373**, Nasonex) is one of the few examples amongst the top selling pharmaceutical drugs that contains a furan ring. Simple furan structures are not normally viable because of their propensity for rapid metabolism by various oxidation mechanisms. Mometasone is a moderately potent glucocorticoid used for the treatment of inflammatory skin disorders, asthma and allergic rhinitis. Because it is delivered topically or is inhaled, it is not subject to rapid metabolism. Structurally, it consists of a chlorinated dexamethasone core which is esterified with 2-furoic acid at the 17-position (**373**, Figure 12).

**Scheme 72:**
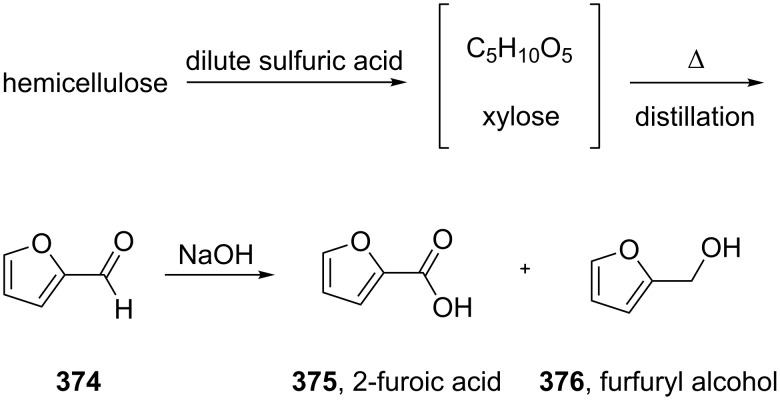
Industrial access to 2-furoic acid present in mometasone.

2-Furoic acid (**375**) can be obtained via a Cannizzaro-type disproportionation of furfural (**374**) which is industrially produced from corncobs: Annual production ca. 450,000 tons. Corncobs contain hemicelluloses which degrade to xylose under acidic conditions. Upon strong heating, xylose is converted to furfural, which can be distilled from the biomass (Scheme 72).

**Scheme 73:**
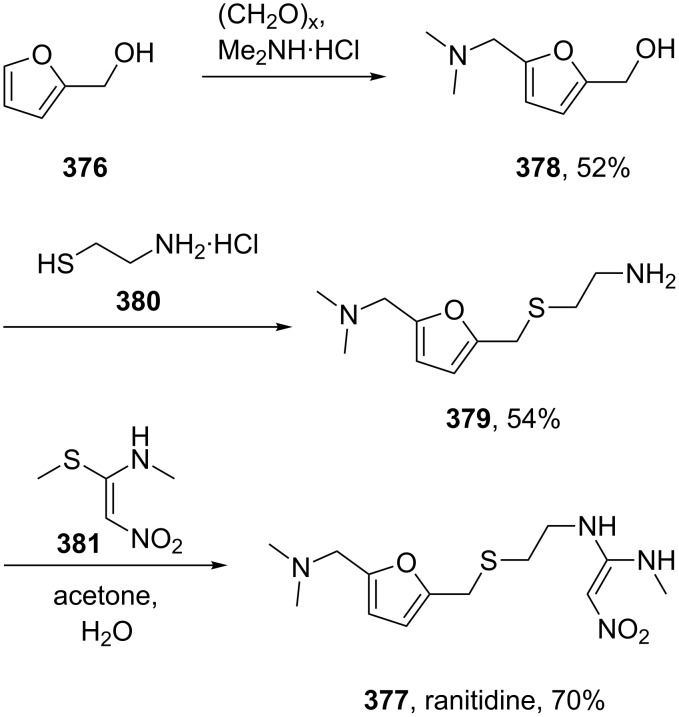
Synthesis of ranitidine from furfuryl alcohol.

The furfuryl alcohol (**376**) obtained in the abovementioned disproportionation (Scheme 72) is also a key starting material for the peptic ulcer therapeutic ranitidine (**377**, Zantac). In the synthesis of this H_2_-receptor antagonist, furfuryl alcohol is subjected to a Mannich reaction with paraformaldehyde and dimethylamine hydrochloride. The resultant (5-((dimethylamino)methyl)furan-2-yl)methanol (**378**) is then treated with cysteamine hydrochloride (**380**) which leads to replacement of the hydroxyl functionality. Finally, coupling of amine **379** with *N*-methyl-1-methylthio-2-nitroethenamine (**381**) furnishes ranitidine (Scheme 73) [112].

**Scheme 74:**
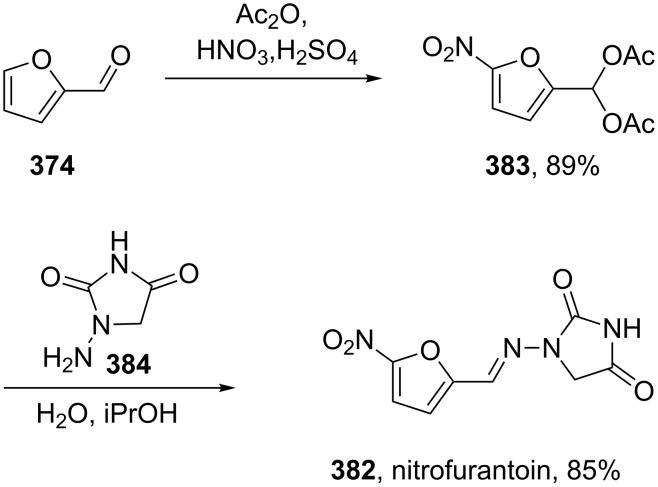
Synthesis of nitrofurantoin.

In addition, the antibiotic nitrofurantoin (**382**, Macrobid) used in the treatment of urinary infections is based on a nitrofurfural building block which can be obtained by nitration of furfural (**374**) [113]. The isolated derivative, acetal **383**, can be converted to nitrofurantoin via condensation with aminohydantoin **384** which itself is obtained from cyclisation of semicarbazidoacetic acid under acidic conditions (Scheme 74) [114].

**Scheme 75:**
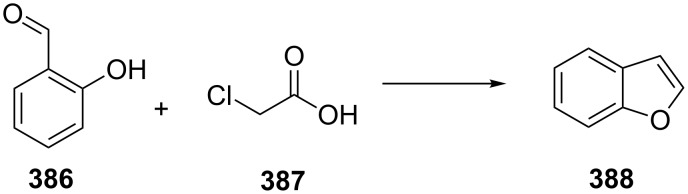
Synthesis of benzofuran.

Amiodarone (**385**, Cordarone) is an antiarrhythmic drug containing a benzofuran ring system. It is one of the most effective antiarrhytmic drugs. Although it is considered a class III antiarrhytmic with its mode of action being principally the blocking of potassium channels, it is anticipated that it is also capable of targeting additional sodium and calcium channels. This might explain its general effectiveness, but could also account for its potentially dangerous side effects. The basic benzofuran framework is commonly prepared by alkylation of salicylic aldehyde (**386**) with chloroacetic acid (**387**) to give the dihydrobenzofuran carboxylic acid which after intramolecular condensation readily decarboxylates (Scheme 75).

**Scheme 76:**
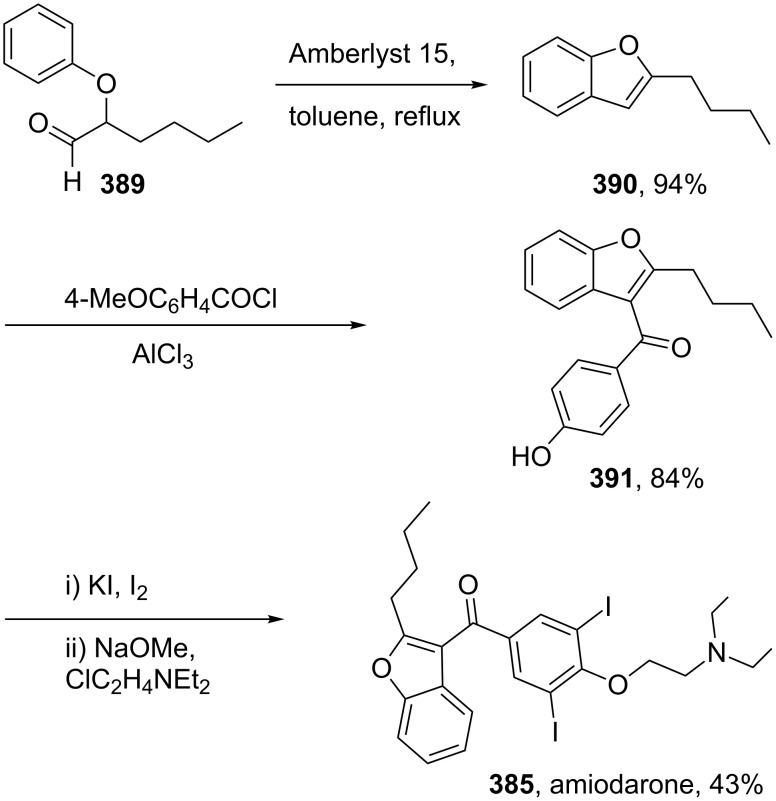
Synthesis of amiodarone.

A more recent synthesis of amiodarone reports the cyclisation of α-phenoxyhexanal **389** under acidic conditions to yield the substituted benzofuran **390** (Scheme 76). A Friedel–Crafts acylation next introduces the aryl ring at the 3-position. Demethylation, iodination and a final alkylation with a diethylaminoethane fragment yields amiodarone [115–117].

### Thiophene

**Scheme 77:**
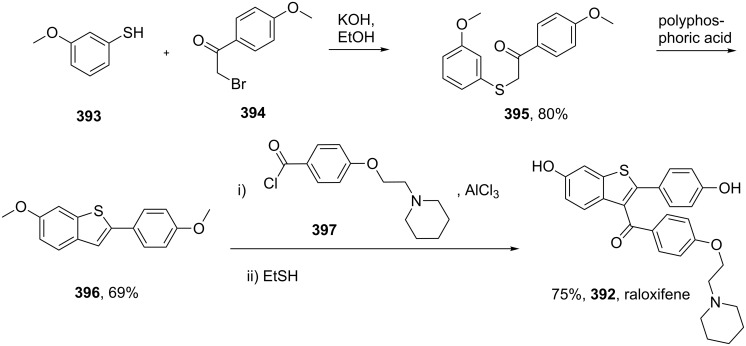
Synthesis of raloxifene.

Considering the fact that thiophene is a classical bioisostere for a benzene ring it is not surprising that it is encountered in many therapeutically active agents. Amongst the drugs containing a thiophene ring are raloxifene (**392**, Evista) and olanzapine (**399** Zyprexa). The former, which is a benzo[*b*]thiophene, is widely used as an oral selective estrogen receptor modulator displaying estrogenic actions on bone (prevention of osteoporosis) and anti-estrogenic actions on breast and uterus, especially in the postmenopausal women [118]. The synthesis of the benzo[*b*]thiophene core **396** was accomplished by condensing 3-mercaptoanisole (**393**) with 4-methoxyphenacyl bromide (**394**) firstly under basic conditions to affect the S_N_2 displacement followed by dehydration with polyphosphoric acid at elevated temperatures (Scheme 77) [119]. Further transformations on this scaffold include the introduction of the carbonyl at the 3-position via a Friedel–Crafts acylation and deprotection, yields raloxifene.

**Scheme 78:**
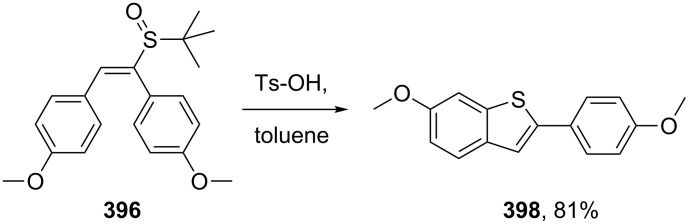
Alternative access to the benzo[*b*]thiophene core of raloxifene.

A non-classical approach to this same benzo[*b*]thiophene core **398** is outlined in Scheme 78 [120]. The vinylic sulfoxide **396** undergoes a formal electrophilic cyclisation under the acidic conditions followed by aromatisation to furnish the desired intermediate in high yield.

**Scheme 79:**
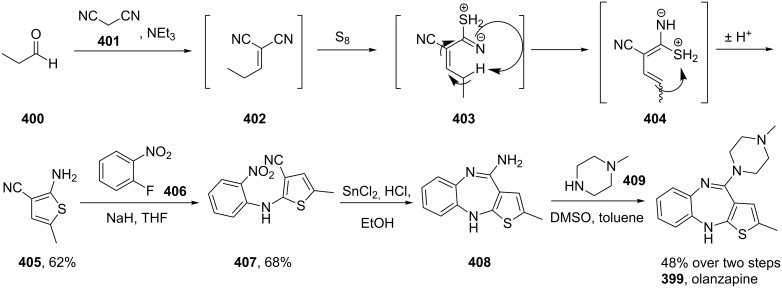
Gewald reaction in the synthesis of olanzapine.

Another atypical antipsychotic drug which has been long established as a top-selling pharmaceutical is olanzapine (**399**) which was first introduced to the market by Eli Lilly in 1996. The thiophene unit is synthesised by a multi-component reaction between malononitrile, elemental sulfur and propionaldehyde (**400**) in the presence of triethylamine. This Gewald-type thiophene synthesis is thought to proceed via an initial Knoevenagel reaction (**400**→**402**) followed by the addition of the sulfur into the nitrile to form thiocyanate **403** which then ring closes to the aminothiophene product **405**. This species is then employed in a nucleophilic aromatic substitution with 2-fluoronitrobenzene (**406**) to give the coupled product **407**. Reduction of the nitro functionality produces the corresponding aniline that readily undergoes ring closure to furnish ultimately the thienobenzodiazepine **408**. Substitution of the pendant amine group with excess *N*-methylpiperazine (**409**) then affords olanzapine in modest overall yield (Scheme 79) [121].

**Scheme 80:**
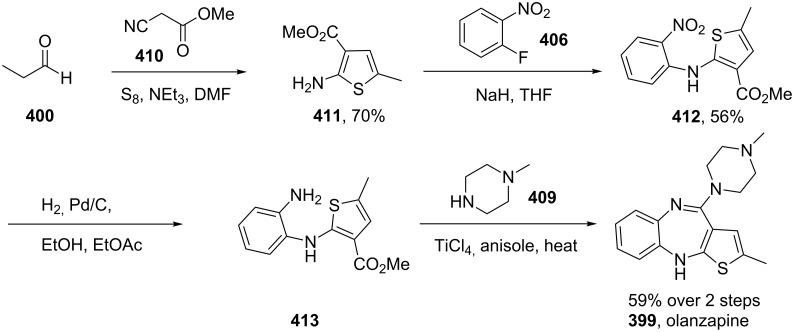
Alternative synthesis of olanzapine.

In a similar process the malononitrile was replaced with the less toxic methyl cyanoacetate (**410**) to give the alternative compound **411** (Scheme 80). The previously used nucleophilic substitution was repeated to furnish the methyl ester analogue **412**. Then, in order to avoid the use of the strongly acidic reduction and cyclisation conditions employed in Scheme 79 (**407**→**408**), a milder palladium catalysed hydrogenation was used. Finally, a one pot intramolecular amide formation followed by a TiCl_4_-mediated reaction to introduce the amidine function yields olanzapine (Scheme 80) [122].

**Figure 13:**
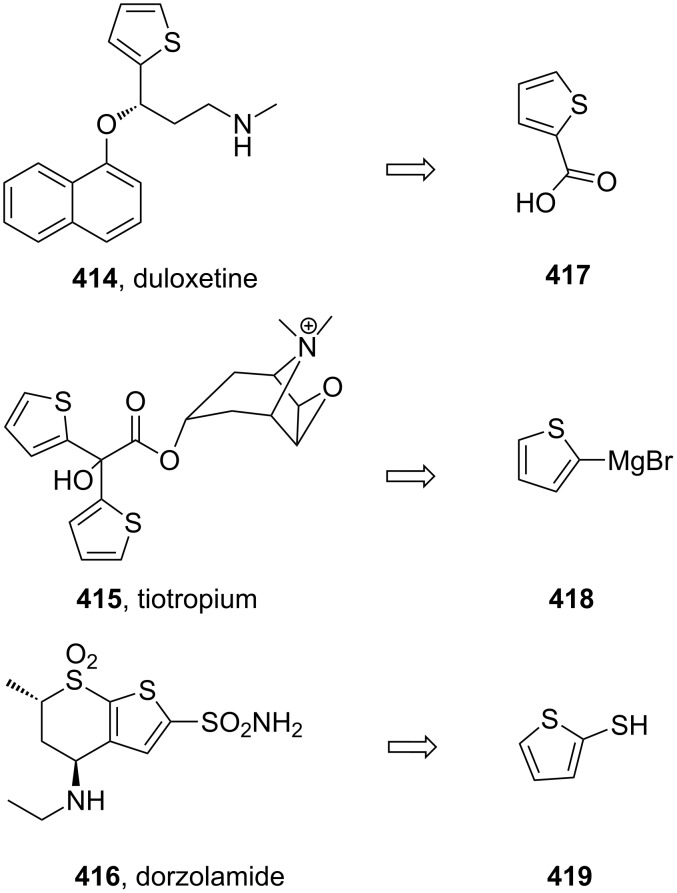
Access to simple thiophene-containing drugs.

Three additional thiophene-containing drugs are duloxetine (**414**, Cymbalta), tiotropium (**415**, Spiriva) and Cosopt [dorzolamide (**416**) (Figure 13) and its second active ingredient timolol (see also Scheme 84)]. However, the syntheses of all three compounds use simple thiophene starting materials, viz. the carboxylic acid **417**, for duloxetine [123], or a Grignard reagent (**418**) for tiotropium [124] and thiophene-2-thiol (**419**) for dorzolamide [125].

**Scheme 81:**
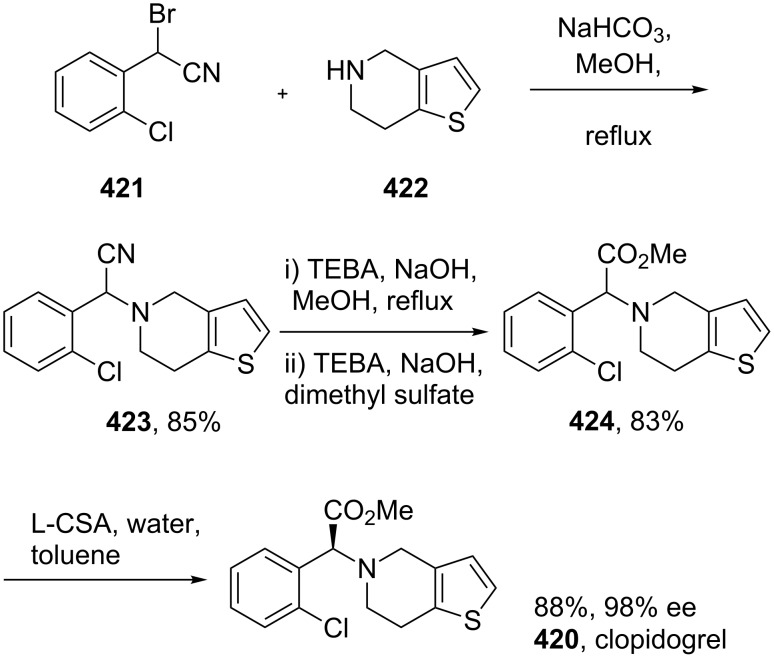
Synthesis of clopidogrel.

One of the most successful platelet aggregation inhibitors currently on the market is clopidogrel (**420**, Plavix) which is a chiral tetrahydrothieno[3,2-*c*]pyridine derivative (Scheme 81). In the favoured synthetic route this tricyclic motif is prepared by a nucleophilic substitution of α-bromo 2-chlorophenyl acetonitrile (**421**) with a secondary amine **422**. Subsequent hydrolysis of the secondary nitrile under phase transfer conditions delivers the free acid which is converted to the methyl ester **424**. In order to obtain the desired *S*-enantiomer, a classical resolution with ca. 0.5 equiv of L-camphorsulfonic acid (L-CSA) in toluene is used. The desired enantiomer **420** is collected as a crystalline salt in greater than 98% ee and 88% of the expected yield (Scheme 81) [126]. The remaining material can be easily epimerised under mildly basic conditions.

**Scheme 82:**
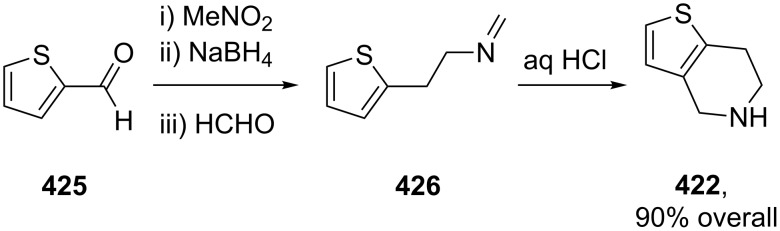
Pictet–Spengler reaction in the preparation of tetrahydrothieno[3,2-*c*]pyridine (**422**).

Although the tetrahydrothieno[3,2-*c*]pyridine structure **422** is now readily available on a large scale from various commercial sources, it was originally synthesised in a variety of ways. The most straightforward route was from thiophene-2-carbaldehyde (**425**) which was subjected to a Henry reaction with nitromethane. Reduction of the nitro olefin function to the corresponding alkylamine followed by reaction with formaldehyde gave the corresponding imine **426** [127]. Treatment of the latter with hydrochloric acid initiates a Pictet–Spengler reaction to furnish the desired heterocycle in high overall yield (Scheme 82).

**Scheme 83:**
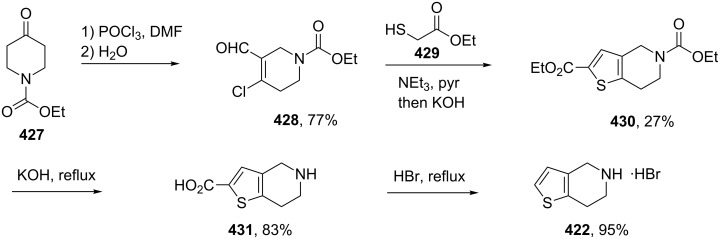
Alternative synthesis of key intermediate **422**.

This key compound can also be accessed by assembling the thiophene ring. In this scenario *N*-protected 4-piperidone **427** is subjected to Vilsmeier conditions to produce the reactive chloroaldehyde species **428** which, upon treatment with ethyl mercaptoacetate (**429**), cyclises to the heterocyclic structure although in only low isolated yield. Simple base hydrolysis of the ester followed by decarboxylation generates the desired product (Scheme 83) [128].

### Thiadiazole

**Figure 14:**
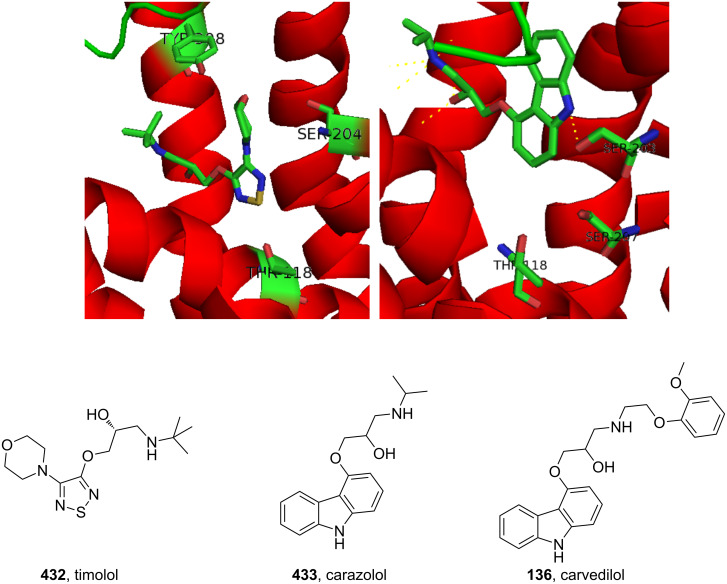
Co-crystal structures of timolol (left) and carazolol (right) in the β-adrenergic receptor.

Cosopt, an ophthalmic medication, consists of the two active ingredients dorzolamide (**416**; Figure 13), a carboanhydrase inhibitor used as an anti-glaucoma agent, and timolol (**432**; Figure 14) a β-adrenergic receptor blocker used to lower intra-ocular pressure by reduction of aqueous humour production. In a recent report an X-ray co-crystal structure of timolol within the β_2_-adrenergic receptor was disclosed [129]. An overlay of this structure with a previous one showing the position of carazolol (**433**), an analogue of the previously mentioned carvedilol (**136**) β-blocker, shows the binding of these molecules (Figure 14). Additionally, this data nicely exemplifies the stronger binding of timolol, as its morpholine group is involved in an extra hydrogen-bonding network with nearby amino acids (Asn, Tyr, Ser) and the thiadiazole motif itself protrudes deeper into the actual binding pocket when compared with the carbazole system of carazolol which results in stronger interactions.

**Scheme 84:**
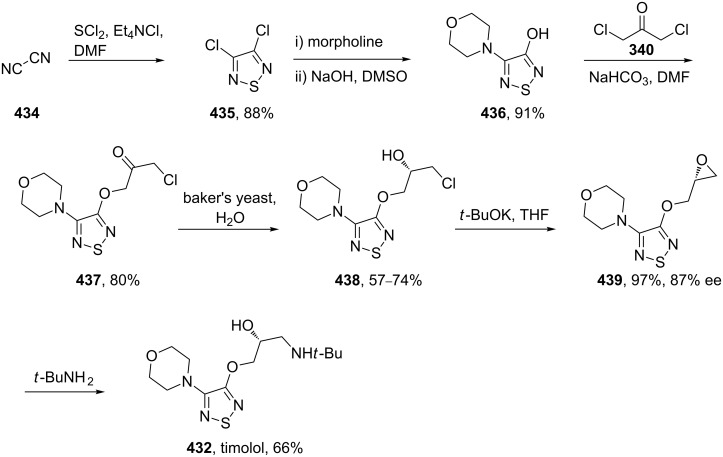
Synthesis of timolol.

Whilst the synthesis of the thiophene-containing component (dorzolamide) of Cosopt starts from 2-mercaptothiophene and elaborates the thienothiopyran motif in a linear multi-step fashion, the thiadiazole ring of timolol is prepared from an acyclic precursor (Scheme 84). The active pharmaceutical ingredient (API) timolol is prepared via a biocatalytic asymmetric reaction which permits selective access to both enantiomers [130]. Starting from 3,4-dichloro-1,2,5-thiadiazole (**435**), which can be prepared from cyanogen (**434**) and sulfur dichloride [131], the two chlorides are differentiated by sequential substitution reactions using morpholine and sodium hydroxide. The resulting hydroxyl in compound **436** is alkylated with dichloroacetone to yield a carbonyl compound **437** susceptible to enzymatic reduction with baker’s yeast. The levorotatory enantiomer thus obtained can be subjected to Mitsunobu conditions with benzoic acid as the nucleophile and leads to clean inversion of the stereocentre which thus gives access to the other enantiomer of timolol. Under basic conditions the chlorohydrin **438** can be converted to the corresponding epoxide which can be ring opened by *tert*-butyl amine to furnish timolol (Scheme 84).

**Scheme 85:**
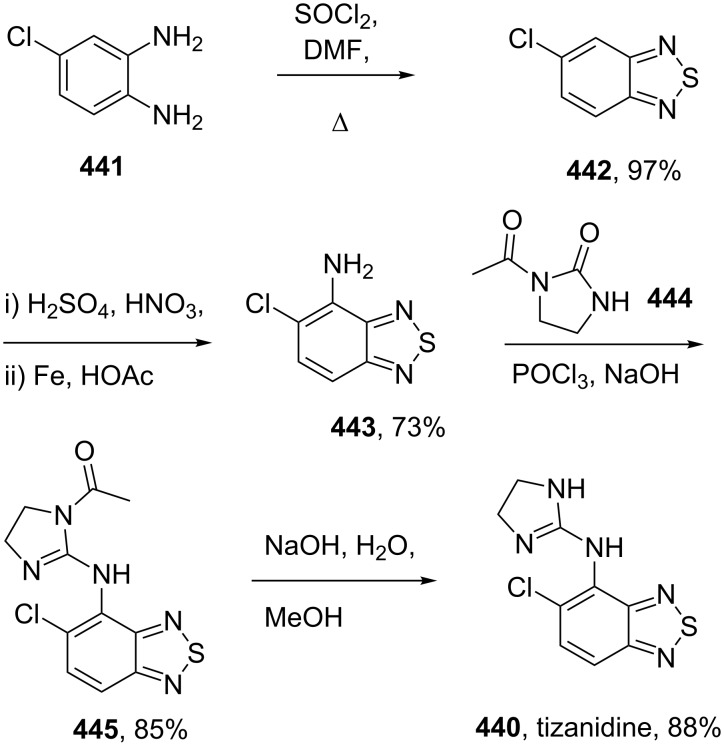
Synthesis of tizanidine **440**.

A related heterocyclic structure can be found in the skeletal muscle relaxant tizanidine (**440**, Zanaflex). This substituted 2,1,3-benzothiadiazole **442** can be prepared by the reaction of an aromatic diamine **441** on heating with thionyl chloride in the presence of DMF. Selective nitration followed by an iron-mediated reduction affords the corresponding aniline **443** which partakes in a nucleophilic substitution of 2-chloro-3,4-dihydroimidazole (generated in situ from the reaction of the urea **444** and phosphoryl chloride). Removal of the acetate group under basic conditions furnishes tizanidine (Scheme 85) [132].

### Isoxazoles and benzisoxazole

The isoxazole ring is a common heterocyclic motif represented by several of the top-selling small molecule pharmaceuticals. This ring structure is often encountered as a surrogate of other nitrogen containing heterocycles such as pyrazoles, pyridines or pyrimidines [133]. Two specific drugs containing this structure are leflunomide (**446**, Arava) and sulfamethoxazole (**447**, Bactrim).

**Scheme 86:**
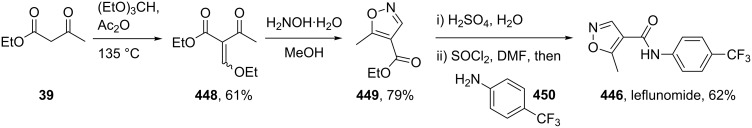
Synthesis of leflunomide.

Leflunomide is a pyrimidine synthase inhibitor of the DMARD-type (disease-modifying anti-rheumatic drug) marketed by Sanofi-Aventis. Unlike NSAIDs, which only deal with symptoms of rheumatoid arthritis, DMARDs target the cause of it. DMARDs are not necessarily structurally or mechanistically related. The effect of leflunomide is possibly due to its regulation of the immune system via affecting lymphocytes. Its synthesis [134] is relatively straightforward starting with a Knoevenagel condensation of ethyl acetoacetate (**39**) and triethyl orthoformate in the presence of acetic anhydride. The resulting ethyl ethoxymethylene acetoacetate (**448**) is next condensed with hydroxylamine hydrate in methanol to yield ethyl 5-methylisoxazole-4-carboxylate (**449**). The ethyl ester is hydrolysed under acidic conditions and the carboxylic acid activated with thionyl chloride in DMF for amide formation with 4-trifluoromethylaniline (**450**) (Scheme 86).

**Scheme 87:**
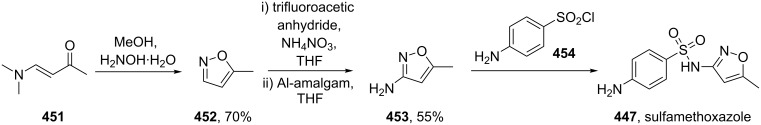
Synthesis of sulfamethoxazole.

A related condensation between (*E*)-4-(dimethylamino)but-3-en-2-one (**451**) and hydroxylamine is used in the synthesis of bactriostatic antibiotic sulfamethoxazole (**447**) to yield the equivalent 5-methylisoxazole (**452**) (Scheme 87). This basic unit is then nitrated with a mixture of ammonium nitrate in trifluoroacetic anhydride **240**, which is presumed to form the active trifluoroacetyl nitrate, and converted to the 3-amino-5-methylisoxazole (**453**) in a aluminium-amalgam mediated reduction [135–136]. Sulfonamide formation with the sulfonyl chloride **454** yields the antibiotic agent **447** (Scheme 87).

**Scheme 88:**
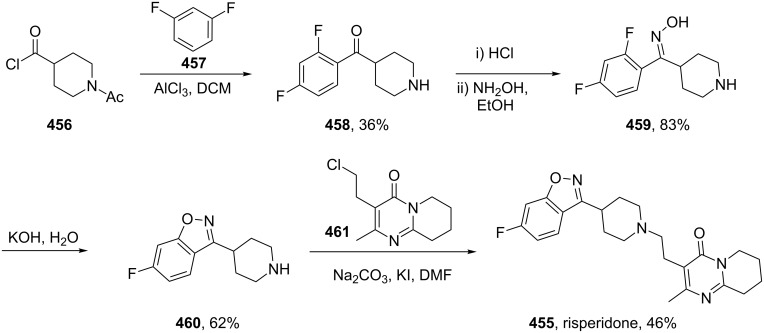
Synthesis of risperidone.

The most prescribed therapeutic for schizophrenia is the benzisoxazole containing antipsychotic risperidone (**455**, Risperdal). For this molecule the benzisoxazole ring is formed via an intramolecular nucleophilic aromatic substitution between an in situ generated oxime **459** and the adjacent aromatic ring [137]. The precursor carbonyl derivative **458** arises from a Friedel–Crafts acylation of the difluoroaromatic **457** (the acetate *N*-protecting group is presumably lost in the work up). Finally, alkylation of the piperidine ring under Finkelstein conditions is used to complete the synthesis of risperidone (Scheme 88).

## Conclusion

**Figure 15:**
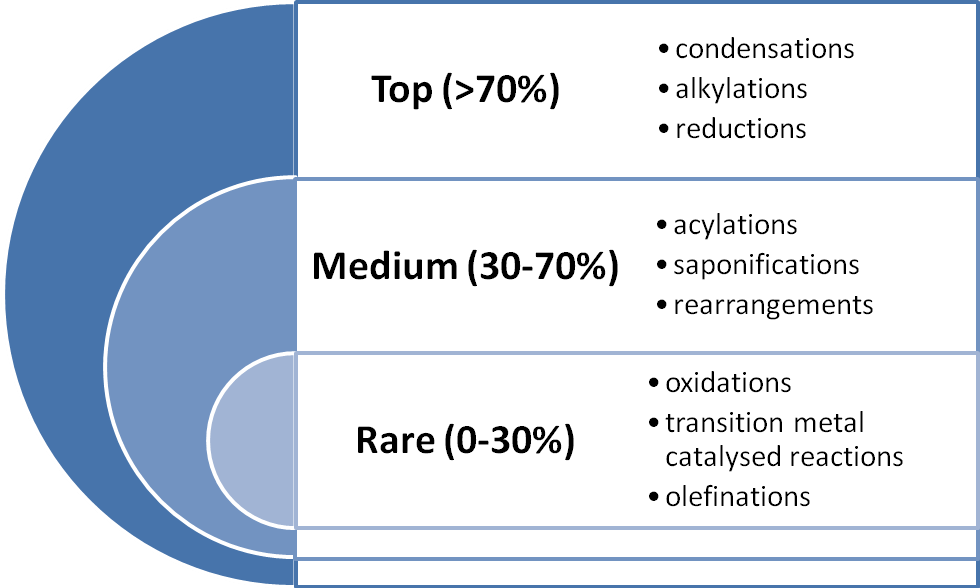
Relative abundance of selected transformations.

Having collected data with respect to many different reported syntheses of drugs containing heteroaromatic five-membered rings, a number of observations can be drawn. For instance, it was established that the most common transformations used are indeed condensations and nucleophilic substitution reactions (Figure 15) [138–139]. This can be rationalised by considering that only small molecular weight by-products such as water or hydrogen chloride/bromide are generated, which are benign and can be easily removed. Other well represented transformations include reductions, amide and ester formations, rearrangements and saponifications, which can be performed in an atom-economic manner based on numerous well established protocols. On the other hand, other robust transformations such as oxidations, nucleophilic aromatic substitutions, olefinations, cycloadditions and metal-mediated transformations appear to be rarely used. The reasons for this might be the need for stoichiometric reagents leading to large amounts of waste as well as potential heavy metal contamination. In addition, these reactions are usually accompanied by the release of heat due to exothermic reaction profiles which might result in difficult to control and therefore undesired chemical processes.

An additional comment should be made about the time frame of new reaction uptake within the process environment. For certain reaction types such as metathesis and C–H activation, which obviously offer significant synthetic potential, it could be argued that the length of time required for these reactions to evolve from research tools to production processes are outwith the pipeline development times of the drugs under discussion. A significant body of the literature representing the synthesis of these drugs date back many years to origins in the late seventies and early eighties. Consequently, it is probably not unexpected to see the absence of many of the ‘newer’ chemical methodologies.

**Figure 16:**
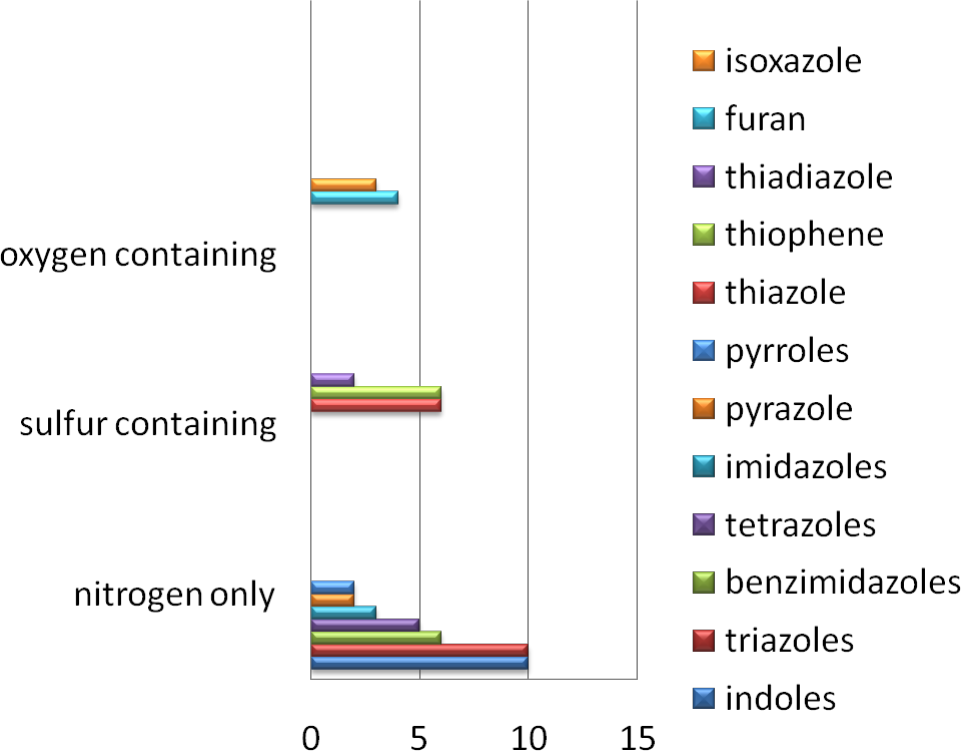
The abundance of heterocycles within top 200 drugs (5-membered rings).

Interestingly, the above-mentioned classes of chemical reactions are most widely applied to a limited number of heterocyclic cores with indoles, triazoles and benzimidazoles being the most prominent examples to date (Figure 16). It can be postulated that this is likely due to two possible factors; 1) the utilisation of so-called ‘me-too drug’ approaches by pharmaceutical companies or 2) the fact that these are privileged structures which avoid the limitations of many other aromatic heterocycles in biological systems such as toxicity. However, the more recent trend seems to indicate a move towards more diverse scaffolds. This might be ascribed to the massive efforts of the pharmaceutical industry to find new drug classes, but could also be an indication of the patenting approaches used to protect more comprehensively these new developments. Furthermore, this move towards novel heterocyclic structures also allows for more flexible substitution patterns permitting extensive SAR studies through efficient high throughput screenings commonly used today.

One constant in all the drug syntheses is the shortness of the reported routes, which typically do not exceed 6–8 steps. Furthermore, chiral centres are still less well represented in drugs unless they can be sourced cheaply from the chiral pool or accessed from commercial suppliers specialised in asymmetric synthesis, for instance, hydrogenations or enzymatic transformations/resolutions. It is predicted [140] that the number of future drugs bearing chirality will increase due to more reliable and affordable catalytic asymmetric transformations [141].

Based on the material presented in this review it can be concluded that most of the reported routes towards well established drugs are based on conventional transformations to furnish the desired compound quickly. However, with increasing competition between the pharmaceutical companies and additional pressures from regulatory/government authorities, chemical producers will need to adopt improved processing techniques that encompass a reduced environmental footprint and deliver the APIs at significantly reduced cost. In order to achieve the necessary productivity gains future chemistry programmes will have to employ a wider range of synthetic transformations targeted at delivering molecules covering an expanded area of chemical space. This will not be solely achieved by minor improvements in the efficiency of individual chemical reactions but will need the industry to integrate actively all the new tools and enabling technologies within their research and manufacturing efforts [142].
